# Self-Rectifying Memristors for Beyond-CMOS Computing: Mechanisms, Materials, and Integration Prospects

**DOI:** 10.1007/s40820-025-02035-1

**Published:** 2026-01-12

**Authors:** Guobin Zhang, Xuemeng Fan, Zijian Wang, Pengtao Li, Zhejia Zhang, Bin Yu, Dawei Gao, Desmond Loke, Shuai Zhong, Qing Wan, Yishu Zhang

**Affiliations:** 1 College of Integrated Circuits, Zhejiang University, Hangzhou, 310027 People’s Republic of China; 2 Yongjiang Laboratory, Ningbo, 315202 People’s Republic of China; 3 Zhejiang ICsprout Semiconductor Co., Ltd, Hangzhou, 310027 People’s Republic of China; 4 ZJU-Hangzhou Global Scientific and Technological Innovation Center, Hangzhou, 310027 People’s Republic of China; 5 Guangdong Institute of Intelligence Science and Technology, Hengqin, 519031 People’s Republic of China; 6 Department of Science, Mathematics, and Technology, Singapore University of Technology and Design, Singapore, 487372 Singapore

**Keywords:** Self-rectifying memristor, Beyond-CMOS, CMOS compatibility, In-memory computing, Neuromorphic computing

## Abstract

The deceleration of Moore’s law and the energy–latency drawbacks of the von Neumann bottleneck have heightened the pursuit for beyond‑CMOS designs that integrate memory and compute. Self‑rectifying memristors (SRMs) have emerged as promising building blocks for high‑performance, low‑power systems by combining resistive switching with intrinsic diode-like behavior. Their unidirectional conduction inhibits sneak‑path currents in crossbar arrays devoid of external selectors, while nonlinear *I*–*V* characteristics, adjustable conductance states, low operating voltages, and rapid switching facilitate efficient vector–matrix operations, neuromorphic plasticity, and hardware security primitives. This review synthesizes the working mechanisms of SRMs, surveys material, and structural strategies and compares device metrics relevant to array‑scale deployment (rectification ratio, nonlinearity, endurance, retention, variability, and operating voltage). We assess SRM-enabled in-memory computing and neuromorphic applications, as well as security functions such as physical unclonable functions and reconfigurable cryptographic primitives. Integration pathways toward CMOS compatibility are analyzed, including back-end-of-line thermal budgets, uniformity, write disturb mitigation, and reliability. Finally, we outline key challenges and opportunities: materials/architecture co‑design, precision analog training, stochasticity control/exploitation, 3D stacking, and standardized benchmarking that can accelerate large‑scale SRM adoption. Through the use of specialized materials and structural optimization, SRMs are set to provide selector‑free, densely integrated, and energy‑efficient hardware for future information processing.

## Highlights

- SRMs integrate intrinsic diode-like rectification, enabling sneak path suppression in crossbar arrays without external selectors, simplifying design, and enhancing energy efficiency for high-density in-memory computing.
- Key metrics such as rectification ratio, nonlinearity, and CMOS compatibility are systematically reviewed, highlighting progress in 3D integration and scalable array.
- Applications span in-memory computing, neuromorphic networks, and hardware security, with emerging potentials in in-sensor computing and self-supervised learning, positioning SRMs as pivotal beyond-CMOS building blocks.

## Graphical abstract

**Figure:**
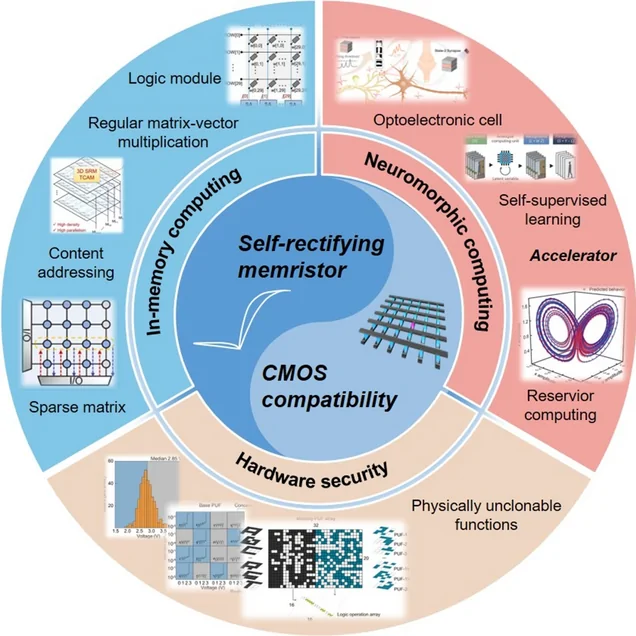

## Introduction

With the rapid advancement of information technology, Moore’s law is increasingly challenged by the physical limits of device miniaturization and rising power consumption issues [1]. Although it has long driven the scaling and performance enhancement of integrated circuits [2], further miniaturization beyond the sub-nanometer regime poses significant hurdles [1, 3]. Technologies such as Fin Field-Effect Transistor (FinFET) have partially mitigated leakage currents [4], yet at the 3-nm node and below, nanosheet gate-all-around (GAA) field-effect transistors are expected to become essential [5, 6]. Moreover, the von Neumann architecture characterized by the physical separation of memory and computing units incurs substantial energy and latency penalties due to continuous data shuttling, thereby limiting overall efficiency [7]. These limitations have spurred interest in beyond-CMOS computing paradigms [8, 9], including in-memory computing and neuromorphic architectures, which merge memory and processing to eliminate data transfer bottlenecks [10, 11]. Neuromorphic computing, in particularly, mimics the structure and functionality of biological neural systems, enabling highly parallel, low-power operation through deep integration of storage and computation.

The realization of ultra-large-scale neuromorphic hardware is essential for emulating brain-like functions in real time and with high energy efficiency, yet it faces critical challenges in maintaining integration density, interconnect complexity, and signal integrity. A critical enabler for such neuromorphic hardware is the passive crossbar array, which offers exceptional scalability and integration density [12, 13]. However, its practicality is hampered by sneak path currents, which impair read/write accuracy. Conventional solutions to mitigate this issue, such as the one-transistor–one-RRAM (1T1R) [14], one-selector–one-RRAM (1S1R) [15], and one-diode–one-RRAM (1D1R) [16] configurations, reduce sneak paths but incur trade-offs in complexity, footprint, power, and variability [12]. An emerging solution is the self-rectifying memristor (SRM), which incorporates inherent diode-like rectification and non-volatile memory within a two-terminal structure. This built-in nonlinearity effectively suppresses sneak currents without external components [17], streamlining design and lowering power consumption. SRMs also exhibit desirable characteristics including high nonlinearity [18, 19], tunable conductance [20, 21], fast switching [22], and low operating power [23, 24], making them a foundational technology for advancing high-density neuromorphic computing systems. While the goal of beyond-CMOS technology is to break the performance limitations of conventional CMOS, in practice, large-scale computing arrays are realized on the premise of compatibility with existing mature CMOS processes [25–27]. This compatibility is an important factor in realizing mass production, cost reduction, and the basis for the smooth integration of new technologies into existing semiconductor manufacturing systems [28]. Therefore, beyond-CMOS technology is faced with an important contradiction to improve system performance while maintaining device compatibility with CMOS processes. Notably, SRMs compatible with CMOS processes have been extensively studied and scaled up to small-scale arrays [17, 29–31]. Meanwhile, SRMs-based in-memory computing architectures and neuromorphic computing systems have been well exploited [12, 17, 32–44], providing an ambitious blueprint for large-scale beyond-CMOS computing paradigm.

**Fig. 1:**
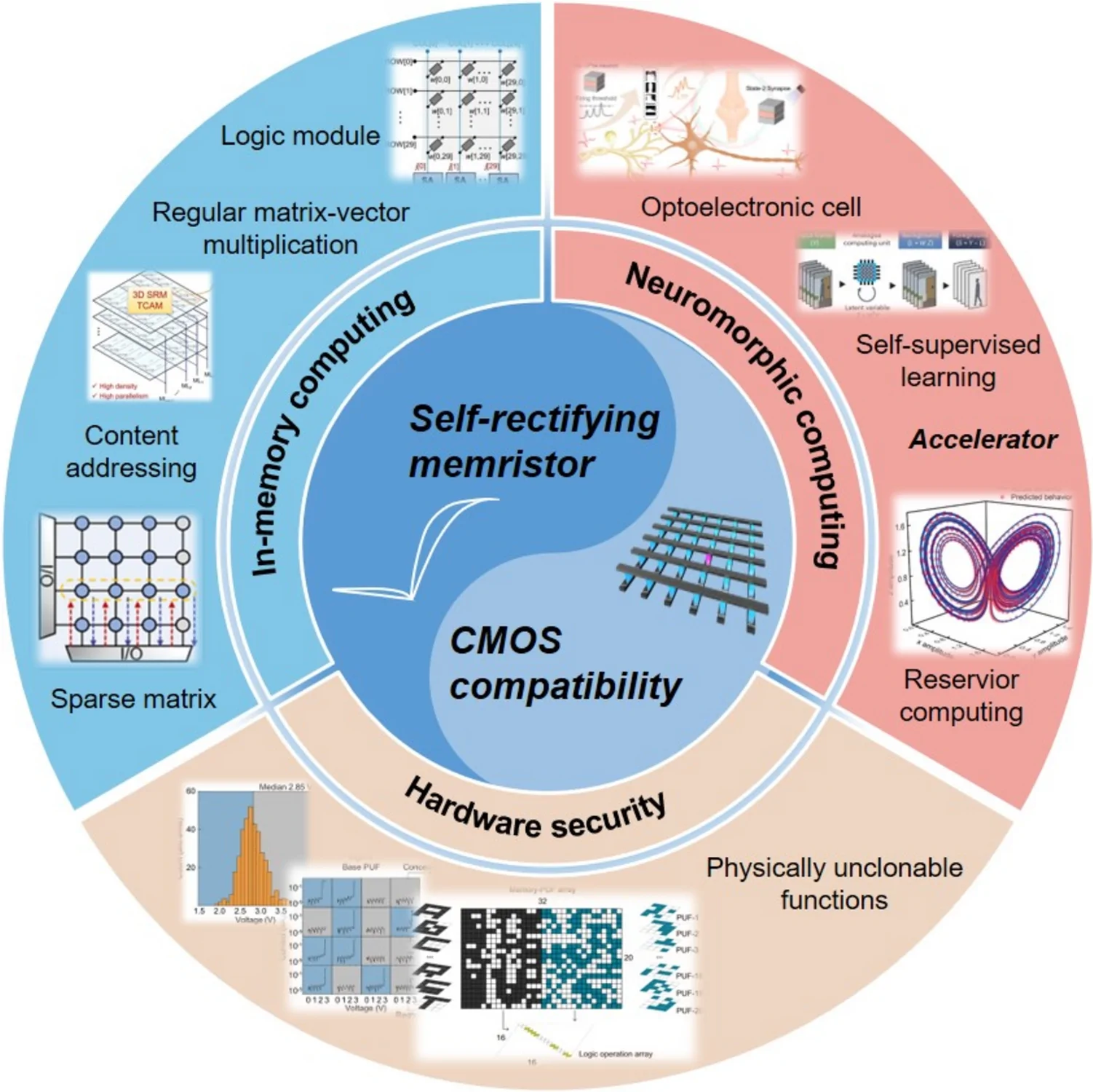
SRMs have been developed for a variety of foreground beyond-CMOS applications, including in-memory computing, neuromorphic computing, and hardware security currently, where CMOS compatibility is an important basis for their further expansion. Reprinted from [17, 34, 37, 45–48], with permission from Springer Nature, American Chemical Society. Copyright 2023 American Association for the Advancement of Science

In this review, we comprehensively examine the potential of SRMs for beyond-CMOS applications, with emphasis on CMOS compatibility and implications for novel computing architectures. Through a systematic analysis of the operating mechanisms, material choices and electrical characteristics of SRMs, we evaluate their advantages and applications in in-memory computing, neuromorphic computing, and hardware security. Finally, the review discusses the prevailing challenges and future opportunities facing the development of CMOS-compatible, high-performance, low-power, and scalable computing systems (Fig. 1). All key terms used in this review and their corresponding definitions are summarized at the end of the document.

## Characteristics and Mechanisms of SRM

### Self-Rectifying Characteristics and Metrics

**Fig. 2:**
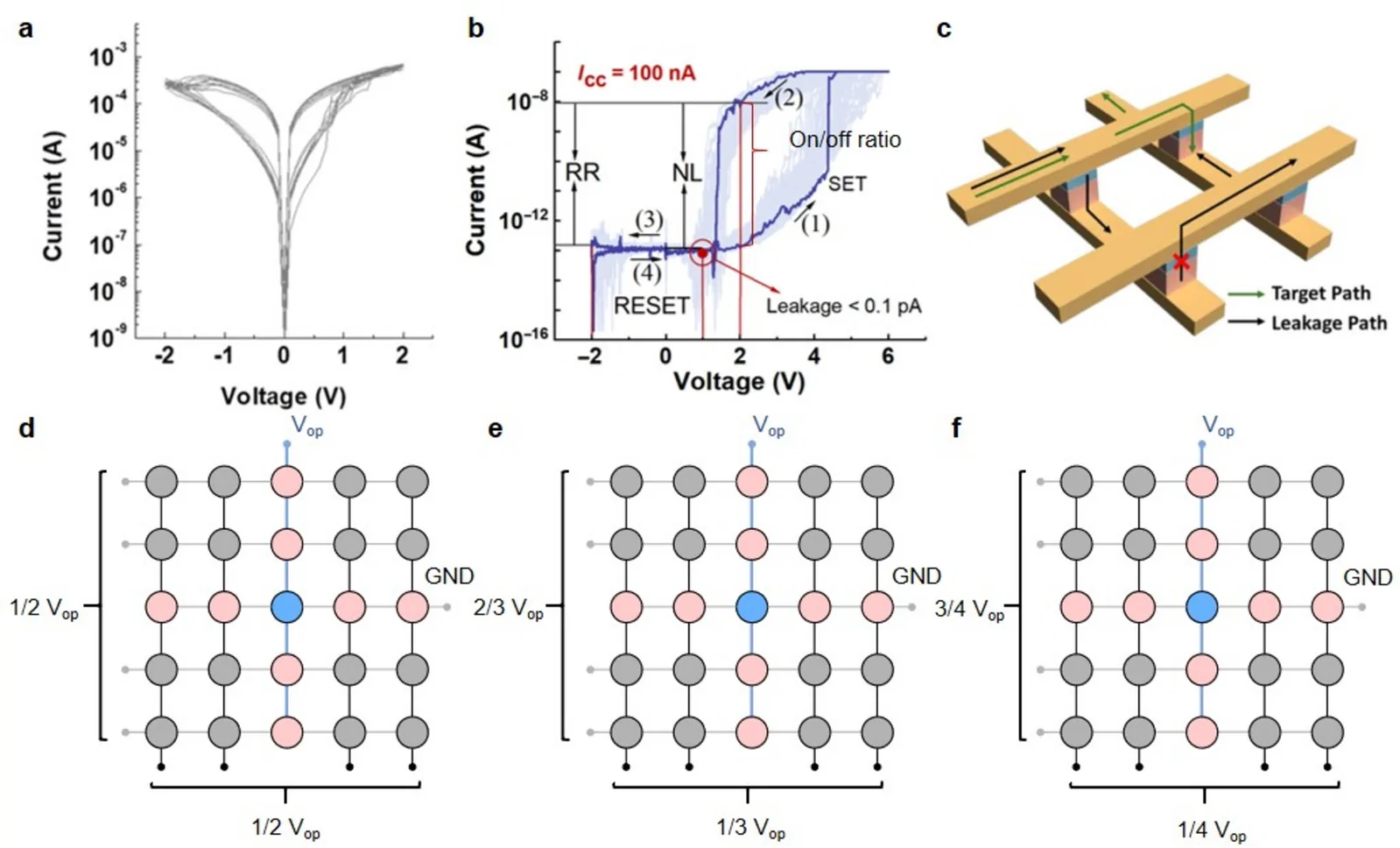
Characteristics of SRMs. **a** Typical DC I–V curves of traditional memristors. Reprinted from [32], with permission of Springer Nature. **b** DC I–V curves of the SRMs based on the structure of Pt/HfO_2_/TaO_x_/Ta. Reproduced from [17], Copyright 2023 American Association for the Advancement of Science. **c** Schematic diagram of the sneak path in the crossbar array consisting of SRMs based on Au/h-BN/Graphene/h-BN/Ag van der Waals heterojunction. Reprinted from [31], Copyright 2024 American Physical Society. The common **d** 1/2, **e** 1/3, and **f** 1/4 voltage scheme when programming SRM cells in the passive crossbar array. The blue cell denotes the selected SRM cell (ideal cell), the pink cells denote the partially selected SRM cells (nonideal cells), and the gray cells denote the unselected SRM cells (nonideal cells)

SRMs exhibit significant differences from conventional memristors in their direct-current current–voltage (DC I–V) characteristics, primarily manifested in enhanced asymmetry and nonlinearity (Fig. 2a, b). These properties stem from deliberate design of the device structure or material interface bandgaps. Conventional memristors typically exhibit symmetric or nearly symmetric I–V loops, with relatively balanced current responses in high- and low-resistance states under positive and negative biases, respectively. This balance facilitates the emergence of “sneak paths” in crossbar arrays. In contrast, SRMs introduce mechanisms such as Schottky barriers, interfacial defect gradients, or asymmetric ion migration. This enables high conduction currents under forward bias while exhibiting strong current suppression under reverse bias, creating a pronounced rectification effect. This self-rectifying characteristic not only effectively suppresses leakage currents but also enables SRMs to achieve high-density integration without requiring external selectors (such as transistors or diodes). In this section, we will comprehensively analyze and summarize the current characteristics and related mechanisms of SRM, and conduct a thorough discussion of its metrics.

#### Rectification Ratio and Nonlinearity

Rectification ratio (RR) is a key parameter describing the performance of SRMs and is defined as the ratio of the on-state current of the device under positive bias to the off-state current under negative bias (Fig. 2b) [17], corresponding to the unselected cells in the crossbar array (Fig. 2c) [31]. Nonlinearity (NL) is defined as the ratio of the current of the device at the read voltage under low-resistance state (LRS) to its current at the half-read voltage (Fig. 2b) with respect to the partially selected cell (Fig. 2c). Both RR and NL determine the level of inter-cell crosstalk in passive crossbar arrays to characterize the accuracy of write and read operations achieved by the array. They reflect the difference in current transfer capability of the device under positive and negative bias and are important parameters of the self-rectifying characteristics. They are both significantly affected by the conduction mechanism. For SRMs, the rectification characteristics mainly originate from the asymmetric structure or interface barriers inside the device. When the upper and lower electrodes of a two-terminal SRM have different figure of merit, a Schottky barrier is formed at the metal/oxide interface, and the difference in the height of this barrier leads to different electron transport characteristics under positive and negative bias. Under positive bias, the barrier decreases and the current passes easily, while under negative bias, the barrier increases and the current is suppressed. In addition, the formation and distribution of the conducting channels affect the rectification ratio. In some SRMs, the conductive channels may be formed or enhanced only under positive bias and weakened or disappeared under negative bias to achieve the rectification behavior. For example, as described above, in ion migration-based SRMs, ions migrate to form conductive channels under positive bias, whereas under negative bias, the direction of ion migration changes, the conductive channels are weakened, and the RR and NL are thus increased significantly [12]. In short, higher RR and NL are highly desirable in SRMs as they significantly enhance array scalability and effectively suppress sneak path currents in crossbar arrays, enabling larger and more reliable passive memory and computing architectures.

#### On/Off Ratio

The on/off ratio of SRM refers to the current or resistance ratio of LRS to high resistance state (HRS) corresponding to its read voltage, which is usually used to measure the degree of resistance change of the memristor under different operating states (Fig. 2b) [17]. This metric is one of the key indicators of the performance of SRMs and traditional memristors, reflecting their switching ability under different resistive states as well as their read margins. The importance of the on/off ratio for SRMs is reflected in several aspects. First, a higher on/off ratio means that there is a more pronounced resistance difference between the LRS and HRS, which contributes to improved signal discrimination and stability. This is critical for applications such as storage and logic operations, as a clear distinction between resistance states reduces misreading and miswriting, thereby improving system reliability and accuracy. Second, a higher on/off ratio helps to reduce power consumption because the leakage current of the device is significantly reduced at high resistive states, which is highly compatible with the purpose of SRM. In addition, in neuromorphic computation, a high on/off ratio can better simulate the weight changes of biological synapses, thus improving the performance of neural networks. Therefore, optimizing the on/off ratio is one of the key directions to enhance the performance and scalability of SRMs and expand their applications [12, 32].

#### Scalability

SRM scalability is the ability to integrate SRMs into large-scale, high-density memory arrays or three-dimensional (3D) integrated architectures while maintaining their critical performance. Due to the intrinsic rectification characteristics of SRMs, the sneak path problem in the array (Fig. 2c) can be effectively suppressed, thus enabling large-scale, high-density integration from two-dimensional (2D) to 3D without adding additional selectors or transistors [14, 15, 41]. Among them, it is worth noting that compatibility with CMOS process is the basis for realizing large-scale SRM-based scaling. Commonly, in the SRM field, read margin is used to characterize the degree of scalability. Read margin is the maximum range or margin of error that can be tolerated during a read operation in a memory or logic circuit. In order to ensure the accuracy of the simulation, it is essential to incorporate RR, NL, and on/off ratio into the calculation of the read margin (Eqs. (1) and (2)) [29]. The read margin is a critical parameter that guarantees the accurate reading of data stored in memory cells, even when the memristor crossbar array is subjected to noise or interference. A higher read margin indicates greater stability and reliability of the crossbar array during data readout, thereby preventing misreading. The one bit-line pull-up strategy is commonly used to calculate the read margin [49].

1 $$\mathrm{RM}= \frac{{V}_{\mathrm{LRS}}-{V}_{\mathrm{HRS}}}{{V}_{\mathrm{pu}}}=\frac{{R}_{\mathrm{pu}}}{{R}_{\mathrm{pu}}+{R}_{\mathrm{s}-\mathrm{LRS}}//{R}_{\mathrm{sneak}}}-\frac{{R}_{\mathrm{pu}}}{{R}_{\mathrm{pu}}+{R}_{\mathrm{s}-\mathrm{HRS}}//{R}_{\mathrm{sneak}}}$$

2 $${R}_{\mathrm{sneak}}=\frac{2\times {R}_{1/3s}}{(N-1)}+\frac{{R}_{\mathrm{uns}}}{{(N-1)}^{2}}$$

Notably, when programming SRM cells, the selection of the voltage scheme directly determines all key metrics including RR, NL, and on/off ratio, thereby impacting the scalability of the SRM and the effectiveness of sneak path suppression. Here, we first consider the 1/2 voltage scheme, as illustrated in Fig. 2d. This scheme applies the full operating voltage (*V*_op_) to the BL where the selected SRM cell resides while grounding the WL, resulting in full positive bias across the SRM cell terminals. Simultaneously, 1/2 *V*_op_ is applied to all other WLs and BLs. In this state, partially selected cells are positively biased at 1/2 *V*_op_, while unselected units remain unbiased [50]. This partially mitigates crosstalk between cells in the crossbar array. Additionally, Fig. 2e, f illustrates the implementation diagrams for the 1/3 and 1/4 voltage schemes, respectively. Only when an optimal trade-off is achieved among key parameters does the selected voltage scheme become meaningful (schemes such as 1/5 or 1/6 may also be considered as appropriate [51]).

**Fig. 3.**
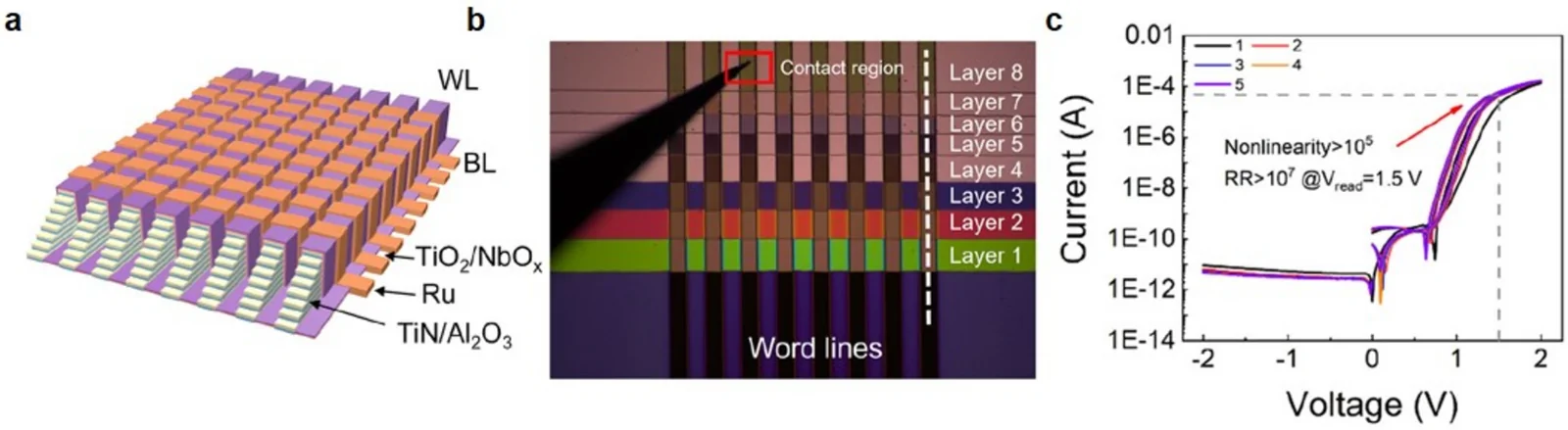
3D integration based on SRMs.** a** Device schematic of the 3D vertical crossbar array based on TiN/TiO_2_/NbO_x_/Ru vertical SRM cell. **b** Optical microscope image of the stepping region from the vertical crossbar array. **c** DC I–V plot of the TiN/TiO_2_/NbO_x_/Ru vertical SRM cell. Reproduced from [49], Copyright 2024 American Chemical Society

Moreover, SRM-based multilayer 3D integration technology represents the critical path to overcoming the density, energy efficiency, and crosstalk limitations of traditional compute-in-memory architectures. Its characteristics of interlayer uniformity, picosecond-level switching energy consumption, and nanosecond-level read latency provide a highly energy-efficient, high-density hardware foundation for complex tasks such as high-precision matrix solving and neuromorphic computing. Li et al. [52] successfully fabricated a 4-layer stacked, 4 Kb-capacity Ta/TaO_x_/HfO_2_/Pt 3D vertical SRM array. Through an innovative “split cell” design, they doubled the integration density and reduced bit cost compared to traditional parallel cell structures. In the fabrication process, multiple pairs of Ta/SiO_2_ layers were alternately stacked using physical vapor deposition and plasma-enhanced chemical vapor deposition. Combined with inductively coupled plasma etching, this formed gate line structures with smooth sidewalls. Uniform HfO_2_ rectifying layers were prepared via atomic layer deposition, ensuring consistency and reliability in the multilayer stacking. This 3D vertical SRM exhibits excellent electrical performance with NL and RR values of approximately 6900 and 4750, respectively. Lu et al. constructed a TiN/TiO_x_/NbO_x_/Ru multilayer stack architecture (Fig. 3a, b) through innovative interlayer isolation and sidewall functional layer deposition techniques [49]. Without external gating devices, its ultra-high RR (> 10^7^) and NL (> 10^5^) effectively suppressed crosstalk currents, enabling 3D SRM arrays to scale beyond 1 Tb (Fig. 3c). Additionally, Ding et al. pioneered a 16-layer 3D vertical SRM [53]. By engineering band structures to form barrier peaks in TiO_x_ and leveraging the low oxygen vacancy aggregation tendency in NbO_x_, they achieved a high NL (> 5000).

### Mechanisms of SRMs

The core of the SRM lies in its simultaneous capabilities of memristive switching and diode-like rectification. Memristive behavior is typically caused by the reversible formation and destruction of mobile ions (such as oxygen vacancies or metal ions), conductive filaments, or the trapping/detrapping behavior of electrons within the material [54, 55]. The transition between the HRS and LRS formed by this process endows the device with multi-state storage capabilities. On the other hand, the rectification function relies on barrier control at the interface layer, where the barrier decreases to allow current flow under forward voltage, while under reverse voltage, the barrier significantly increases to limit current, thus creating directional conduction characteristics. Generally, the operating principle of SRMs is determined by the combined effects of material properties and structural design. In terms of material selection, functional materials with ionic migration characteristics, such as oxides and sulfides, are commonly used; in structural design, heterojunctions or asymmetry between the electrodes and the active layer form the basis for rectifying behavior. A deep understanding of this mechanism not only helps to enhance device performance but also provides an important theoretical basis for the development of new types of memory devices. Below is a detailed introduction to the mechanism including interface barrier (Schottky effect, interface oxygen vacancies), ionic migration, and trap effects (oxygen vacancies).

#### Interface Barrier

**Fig. 4:**
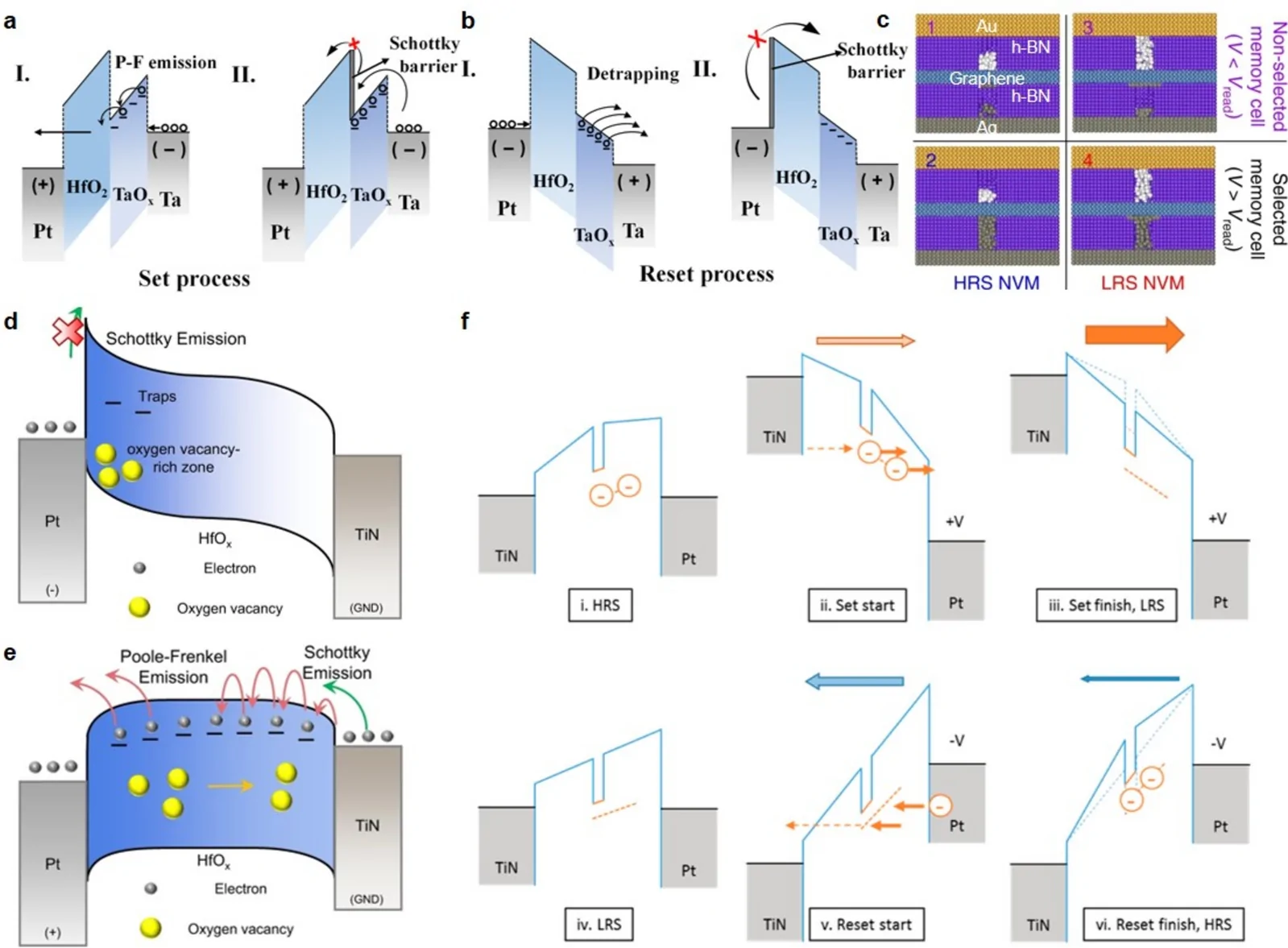
Mechanisms of SRMs. **a** Conductivity mechanism of double-layer oxide-based SRMs under positive bias and **b** positive bias. Reproduced from [17], Copyright 2023 American Association for the Advancement of Science. **c** Conductivity mechanism of SRMs based on Au/h-BN/graphene/h-BN/Ag van der Waals heterojunction. Reproduced from [41], with permission from Springer Nature. The conducting mechanisms, including interfacial barrier and ion migrant, of the proposed SRMs when **d** negative bias and **e** positive bias are added, respectively. Reproduced from [51], with permission of Springer Nature. **f** Illustration of the charge trapping and detrapping processes in the SRM cell based on Pt/NbO_x_/TiO_y_/NbO_x_/TiN structure. Reprinted from [43], Copyright 2016 American Chemical Society

The core role of the interface barrier in SRMs lies in introducing asymmetric charge conduction characteristics through physical mechanisms (Schottky barrier, tunneling effect). By reasonably selecting electrode materials, regulating interface chemical properties, and utilizing the defect distribution in oxide films, the barrier height and rectification performance can be conveniently adjusted. The rectification function of SRM is primarily caused by the different Schottky barrier heights between the two electrodes and the functional layer (Fig. 4a) [17].

Li et al. reported a p-Si/SiO_2_/n-Si memristor, where an asymmetric barrier exists at the Si/SiO_2_ interface (p–n junction effect) [56]. The modulation of the electric field facilitates the formation of carrier transport paths under forward bias, while suppressing leakage current under reverse bias, thus generating self-rectifying behavior with an excellent rectification ratio (10^5^) and good retention performance (> 2 × 10^5^ s). Similar to self-rectifying devices such as Pt/TiO_x_/Ti and Al/MoO_x_/Pt, Ni et al. reported a Pt/TaO_x_/Ta memristor, where the combination of low work function and high work function electrodes forms an asymmetric Schottky barrier, achieving directional rectification [57]. The functional layer (TaO_x_) acts as a switching medium, supporting HRS and LRS transitions and memory functions by regulating the internal distribution of oxygen vacancies, exhibiting superior rectification ratio and nonlinearity. Most reliable SRMs benefit from the blocking layer that restricts the disordered diffusion of charge carriers, preventing the formation of complete conductive filaments, which is a cause of leakage current. The presence of the blocking layer effectively suppresses the leakage current of the material under low voltage or reverse bias, thereby enhancing the reliability of device writing and reading. Additionally, the blocking layer can reduce power consumption, as the lower leakage current implies a more energy-efficient operating state. In numerous studies, the blocking layer forms a thin barrier, limiting the migration of ions/electrons, executing tunneling/thermionic emission mechanisms. As a result, the device's nonlinearity, durability, and data retention capability are improved. In 2023, Li et al. [17] proposed a SRM based on the Pt/HfO_2_/TaO_x_/Ta structure, where HfO_2_ serves as the blocking layer. Under positive bias, electrons migrate through shallow energy level defects in the functional layer, with Poole–Frenkel (P–F) transport dominating the barrier crossing in the switching layer (Fig. 4a). When the positive voltage decreases to a certain value, the electron energy is insufficient to overcome this high barrier. The interruption of electron transport returns to the HRS, exhibiting high nonlinearity. Under negative voltage, a Schottky barrier forms between Pt and the blocking layer HfO_2_, blocking electron conduction between the electrodes and resulting in low current, leading to the rectifying effect observed in the device (Fig. 4b). The DC I–V curves of typical SRMs are shown in Fig. 2b.

Zhang et al. reported a Pt/HfO_2_/WO_x_/TiN SRM structure, where the abundant traps in the WO_x_ switching layer and the excellent insulating properties of HfO_2_ synergistically promote positive polarity while suppressing negative polarity current, achieving a rectification ratio exceeding 10^6^ [29]. Meanwhile, the increase in the thickness of the switching layer leads to a higher number of defects, resulting in a greater probability of electrons being trapped after passing through the switching layer. Under negative bias, the number of electrons emitted from traps decreases, leading to smaller leakage current. Conversely, under positive bias, more trapped electrons favor current accumulation, generating larger positive current. Lee et al. proposed a SRM based on the Ti/NiO_x_/Al_2_O_3_/Pt structure [58]. Under positive bias, the migration of oxygen vacancies from the NiO_x_ RS layer to the Al_2_O_3_ blocking layer reduces the barrier thickness in the Al_2_O_3_ blocking layer, facilitating tunneling to produce high current values. Under negative voltage, the Schottky barrier at the Ti/NbO_x_ interface and the thickening barrier of the Al_2_O_3_ rectifying layer jointly suppress reverse leakage current. In 2025, Pham et al. conducted an in-depth analysis of the underlying mechanism of interfacial conduction in SRM from the perspective of band theory, making significant contributions to the advancement of this field [59]. Similarly, the HfO_x_/ZrO_y_ structure also presented dominant interfacial mechanism recently [60]. These studies indicate that the self-rectifying properties are not solely determined by the material work function; the generation, distribution, and migration of ions also affect the barrier thickness and energy band height.

#### Ion Migration

Ion movement-type memristors utilize the migration and distribution of active ions (such as metal cations or oxygen vacancies) under an electric field to regulate the device's resistance state. Through specially designed device structures (such as gradient oxide layers), ion movement can be promoted in one direction to form conductive channels (low resistance) while being suppressed in the opposite direction (high resistance), thereby achieving the self-rectifying characteristics of the current (Fig. 4c) [41].

Kim et al. fabricated a Pt/Na-doped TiO_2_/Pt-based SRM, where the asymmetric distribution of defects regulated the migration of Na ions, leading to self-rectifying behavior [36]. Lim et al. proposed alkali metal ion SRMs, utilizing lithium metal as the adhesive layer for the bottom electrode, with an alkali ion reservoir forming at the bottom of the memristor layer [61]. Aluminum dopants were used to improve retention characteristics and suppress the diffusion of alkali cations. In the optimized aluminum-doped memristor device, retention characteristics were maintained for over 20 h at 125 °C, durability exceeded 5.5 × 10^5^ cycles, and high linear/symmetrical weight update characteristics were achieved.

Bae et al. developed a fluorine ion-doped TiO_x_ SRM [42]. Fluorine ions attracted oxygen vacancies, reducing the migration energy of nearby oxygen vacancies, which improved the reversible redistribution and reduced device variability. The fluorinated memristor showed improvements in switching ratio, rectification ratio, device time uniformity, and switching speed, overcoming the trade-off between performance and reliability seen in traditional memristors. Zhang et al. reported Ni-doped WO_x_/ZnO SRMs, where Ni ions reduced the electron affinity of the WO_x_ layer, forming asymmetric electron transport paths with shallow deep-level traps, leading to self-rectifying behavior [30]. Interestingly, the concentration gradient caused an asymmetric distribution of ions within the device, creating an internal electric field. The direction of this electric field either reinforced or weakened the applied bias direction, altering the switching bias between the on-state and off-state, resulting in nonlinear differences in conductivity under two polarities. To achieve unique dynamic functions with large-scale in-memory computing, Choi et al. fabricated dynamic Pt/WO_x_/W SRMs [45]. The asymmetric distribution of oxygen vacancies in WO_x_ between the Pt and W electrodes led to the formation of a stable Schottky barrier at the Pt/WO_x_ interface and dynamic modulation of the Schottky barrier at the WO_x_/W interface. The migration of oxygen vacancies driven by the electric field was observed even without externally applied compliance current, exhibiting high switching uniformity and device yield (> 98%), stable durability (> 10^5^ cycles), and low programming voltage (~ 0.7 V) for self-rectifying switching.

Wang et al. proposed an SRM structure of Pt/WO_3_/WO_3-x_/TiN [31]. The concentration of oxygen vacancies in the WO_x_ functional layer exhibited a gradient distribution, causing different energy level distributions at the top and bottom electrodes, resulting in asymmetric energy barriers for electron movement. This achieved a remarkable rectification ratio (approximately 10^5^), a satisfactory switching ratio (approximately 10^3^), low operating voltage (2 V), and high stability (> 10^6^ s, 10^4^ cycles). When integrated into a 100 × 100 array, the device achieved a significant resistance reading accuracy of 97.3%. Additionally, by setting the read margin at 10%, the passive array integrated with this device could reach a storage capacity of up to 180.3 Gb. Zhang et al. proposed Pt/NiO_x_/WO_3−x_:Ti/W self-rectifying devices, where the difference in work function between the rich O^2−^ region at the NiO_x_/WO_3-x_:Ti interface and the rich region of oxygen vacancies at the WO_3-x_:Ti side formed an interface barrier, resulting in HRS and low conductivity [33]. By controlling the oxidation in the two oxide layers, the HRS current and interface barrier could be optimized, achieving ultra-high weight-enhanced linearity (0.9997). Choi et al. proposed TaO_y_/NP TaO_x_ SRMs, where the device operation relies on the formation, movement, and aggregation of oxygen vacancies in the nanopore structure [62]. When a certain electric field is applied, the migration of oxygen vacancies changes the resistance state of TaO_x_ from the HRS to the LRS, realizing memristive behavior. The self-rectifying characteristics are caused by asymmetric conduction induced by the heterojunction between the TaO_y_ thin film and the nanoporous TaO_x_ layer, achieving low-power, stable, and interference-resistant memristive switching behavior. Sun et al. [41] proposed a self-selective storage unit based on an Au/h-BN/Graphene/h-BN/Ag van der Waals heterojunction, where h-BN and graphene represent hexagonal boron nitride and graphene, respectively. Non-volatile boron vacancy layers and volatile silver layers were formed in the Au/h-BN/ Graphene and Graphene /h-BN/Ag structures (Fig. 4c). In the unit integrating non-volatile and volatile structures, the graphene layer effectively prevented the diffusion of the volatile silver layer, resulting in highly nonlinear resistance switching with self-selection of 10^10^ and a switching resistance ratio exceeding 10^3^. Further, as illustrated in Fig. 4d, e, combining interfacial barrier and ion migrant could realize surprising performance of SRMs, exemplified by the Pt/HfO_x_/TiN single-layer SRM [51].

#### Defect Regulation

The resistive switching characteristics of devices are due to the capture and release of charge carriers (such as oxygen vacancies, metal ions, or other defect states), while the rectifying characteristics arise from controlling the filling and release directionality of charge carriers.

Kim et al. designed a low-current self-rectifying Pt/NbO_x_/TiO_y_/NbO_x_/TiN device, where the memristive behavior is attributed to the electron capture/detrapping process, and the asymmetric barrier results in a self-rectification ratio of 10^5^, with trap energy levels formed up to 0.8 eV in the TiO_y_ layer (Fig. 4f) [43]. By optimizing the dynamic behavior of oxygen vacancies in the active layer and the design of the barrier layer, excellent performance was further achieved through using of ion migration driven by local electric fields and built-in electric fields at heterogeneous interfaces: no forming process required, self-rectification, high rectification ratio, low-power operation, and asymmetric programming voltage. Yoon et al. prepared Ta_2_O_5_/HfO_2-x_ based SRMs, where Ta_2_O_5_ with high electron affinity serves as the rectifying layer [44]. Although this design weakens the Schottky barrier at the rectifying interface, it facilitates the forward injection of electrons within the dielectric layer. Cheong et al. reported a Pt/HfO_2_/Nb_2_O_5_/HfO_2_/Ti SRM, with the Nb_2_O_5_ layer acting as a charge trap layer [63]. Due to the oxygen scavenging effect of the bottom Ti electrode, even with the same HfO_2_ layer, the lower HfO_2_ film contains oxygen defects, which aids in trap-assisted tunneling.

Ionic memristors, relying on the dynamic migration of oxygen vacancies/ions, exhibit excellent dynamic control capabilities and multi-resistive state storage characteristics, making them suitable for online learning and neuromorphic computing, but they have poor long-term stability and complex fabrication processes. Barrier-type memristors provide high rectification ratios and stability through interface barrier engineering, making them suitable for leakage current suppression and high-density storage, but they lack dynamic control capabilities and have lower complexity, which can be optimized through multiple metal–oxide systems. Defect-type memristors, based on the distribution of material defects, offer stable switching performance and simple manufacturing processes, with lower power consumption than barrier-type memristors, making them suitable for fixed-weight storage, but their rectification ratios and dynamics are relatively limited. The choice among these three mechanisms depends on the requirements of the application scenarios.

### Metrics

In this section, we delve into the key factors influencing the core device-level metrics of SRMs—such as RR, nonlinearity NL, CMOS compatibility, switching speed, and reliability—which have been partially introduced previously. The discussion will focus on how material selection, interface engineering, structural design, and switching mechanisms affect these critical performance parameters. By analyzing these influencing factors, we aim to provide deeper insight into the operational principles and performance limits of SRMs at the device level. A comprehensive review of array-level implementations and system applications will be presented in subsequent sections.

#### Rectification Ratio

As mentioned earlier, the RR of SRMs is closely related to the energy band structure of the selected electrodes, resistive and insulating layers. For example, oxygen anion migration and barrier modulation can improve the rectification characteristics of TaO_x_/Al_2_O_3_ memristors [64], but optimizing the thickness of the Al_2_O_3_ switching layer requires precise control of the distribution and migration of the oxygen vacancies to achieve the expected RR. For material design, it is not the case that simply increasing the barriers of the metal-insulating layer can lead to a larger RR, but also the contact barriers of the metal–oxide at the other end as well as oxide–oxide and the state of the individual interfaces need to be considered. If the barrier between the metal-insulating layer is too large, the emitted electrons cannot tunnel through the insulating layer to the other end, thus suppressing the positive current and further not achieving a sufficient RR. In addition, the optimization requires precise control of the preparation process parameters, such as temperature, time, atmosphere, etc. When the resistive layer film needs to be prepared, it is necessary to control the temperature, time, atmosphere, etc. precisely. When the resistive layer films need to be annealed rapidly in argon or oxygen atmosphere, the increase in oxygen vacancies leads to changes in the nature of the interfacial contacts, which affects the rectification effect. Since the roughness, defect concentration, and other factors of the films at different locations are completely different, precise control of such process parameters is difficult to achieve in large-scale production, significantly increasing the difficulty of optimizing RR.

#### Nonlinearity

Unlike RR, NL is mainly influenced by the first metal–oxide barrier in the conducting direction, and a proper barrier will result in a wide NL region corresponding to the SRM [17]. Optimizing NL encounters the same challenges as RR, where excessive metal–oxide and oxide–oxide barriers can similarly limit the magnitude of the peak current and thus the NL enhancement. Also, when there are too many defects such as oxygen vacancies in the resistive layer, the defects will continue to migrate and accumulate with the small electric field and thus form conductive filaments, at which point the current, which would otherwise barely increase with increasing voltage, will gradually rise, i.e., NL failure [30].

#### On/Off Ratio

The on/off ratio needs to be synergistically optimized with RR and NL rather than sacrificed. First, the energy band structure and oxygen vacancy concentration have a direct effect on the on/off ratio. For example, the increase in oxygen vacancies in the WO_3-x_ layer improves the electron trapping and de-trapping efficiencies, thus increasing the on/off ratio [31]. Subsequently, preparation process parameters such as temperature, time, and atmosphere have important effects on the energy band structure and oxygen vacancy distribution of the materials, e.g., the preparation of WO_3_ and WO_3-x_ layers by low-power and high-power sputtering techniques, respectively, achieves different oxygen vacancy concentrations, which in turn affect the on/off ratio [31]. Similarly, the selection of electrode materials and intermediate oxide layers has a significant effect on the on/off ratio, e.g., in the Pt/WO_3_/WO_3-x_/TiN structure, the difference in the work function between the Pt and TiN electrodes and the WO_3_ layer, as well as the oxygen vacancy traps in the WO_3-x_ layer, works together to achieve a high on/off ratio [31]. Wang et al. systematically verified the above phenomena and mechanisms to achieve a large on/off ratio while realizing perfect nonvolatility [31], providing a fundamental guide for large-scale scaling. However, the blind pursuit of large on/off ratios may sacrifice the data retention capability of SRMs [40], which in turn fails to store data reliably, which is unnecessary.

#### Switching Speed

The optimization of the switching speed of SRMs is limited by several factors. First, SRMs usually rely on materials with nonlinear ion mobility properties, and the ion mobility rate and defect distribution of these materials directly affect the device response speed. Although few SRMs have a conductive mechanism based on conductive filaments [41], continuous conductive pathways formed by defects such as oxygen vacancies can also prevent devices from switching (conductive pathway formation is slow) [29, 31, 47]. Second, the design of the device structure has a significant impact on the switching speed; the interfacial properties between the electrodes and the memristive material, the device dimensions, and the homogeneity of the internal electric field distribution all affect the efficiency and path of ion migration, thus constraining the optimization of the switching speed. Ultra-thin oxide resistive and insulating layers enable ultrafast switching [17, 49], while increasing the thickness above 30 nm significantly slows down the switching significantly [47]. In addition, external operating conditions such as voltage amplitude, pulse frequency, and temperature can also have a significant impact on switching speed; too high or too low a voltage can lead to material damage or impeded ion migration. Even though Lu et al. achieved ultrafast response at the ps level, the overly complex preparation process based on 16 layers limits its potential for large scaling [49]. Nevertheless, they still provided valuable instructions concerning moderate 3D integration for fast switching with ultra-thin film.

Optimizing for a higher RR or NL often involves increasing the barrier height at the device interfaces. While this can effectively suppress reverse currents, it may also increase the energy barrier for electron transport under forward bias, thereby slowing down the switching speed [65]. For example, in devices with high RR and NL, the formation and rupture of conductive filaments may require higher activation energies, leading to longer switching times.

#### CMOS Compatibility

Recently, combined with Ag⁺-mediated filamentary switching in the 2D perovskite channels, the design from Son et al. overcomes the voltage-consumption trade-off plaguing conventional SRMs [66]. However, constructing high-performance SRMs based on 2D materials that are incompatible with CMOS processes contradicts the path of future development. There are numerous CMOS process-compatible resistive layer oxides used for SRMs, and the one that has been extensively studied is WO_3_ [29–31, 45]. WO_3_ has abundant oxygen vacancies and tunable conductive properties, and these oxygen vacancies are able to migrate under the action of an external electric field to form or break conductive filaments and achieve the resistive behavior [67, 68]. At the same time, the high thermal and chemical stability makes it perfectly compatible with CMOS processes. However, the compatibility of other mainstream resist materials with CMOS processes still needs to be further explored, for example, indium–gallium–zinc–oxide (IGZO) has excellent conductivity tunability, electron mobility, and photographic properties [69], but high temperature oxidation or annealing environments in the CMOS process can lead to the denaturation of the IGZO film. Although popular and with good CMOS compatibility, the resistive denaturation mechanism of WO_3_ relies on the migration of oxygen vacancies and the formation of conductive filaments, and its oxygen vacancies are poorly controllable, which therefore tends to lead to instability and poor uniformity of SRM performance. Therefore, the development of CMOS process compatibility of other resistive oxides is a major challenge but a necessary path to achieve large-scale integration. High-k oxides, such as HfO_2_ [17], Ta_2_O_5_ [44], Al_2_O_3_ [70], TiO_2_ [46], and others with good CMOS compatibility, have also received much attention. For example, notably, the SRMs proposed by Zhang et al. exhibit CMOS middle-of-line compatibility, leveraging HfO_2_ and TiN—materials routinely integrated in intermediate fabrication stages [29], and the architecture present by Wang et al. demonstrates back-end-of-line process alignment, where Pt electrodes and WO_3_ layers conform to back-end metallization requirements [31]. These distinct material stacks reflect tailored integration strategies for CMOS workflows, respectively.

#### Reliability

The reliability characterization of SRM is consistent with that of common memristor with data retention and endurance as the two main factors [71]. As shown in Table 1, starting from 2015 until 2025, SRMs have experienced a gradual development from data retention characteristics that are generally less than 10^4^ s to greater than 10^4^ s or even resistant high temperature. Endurance follows a similar pattern of development. It is worth noting that reliability is very closely linked to operating voltage. High operating voltage leads to a significant increase in the electric field strength inside the SRM, accelerating the migration of oxygen vacancies or metal ions, thus promoting the formation or breakage of conductive filaments. But the ion migration under this strong electric field is often difficult to be precisely controlled, which easily triggers excessive or non-uniform filament growth and leads to unstable device performance. In addition, high operating voltages can exacerbate the Joule heating effect within the material, and the localized temperature increase may cause structural changes (e.g., crystallization or phase transitions) in the material, or even lead to chemical reactions or degradation at the interface between the electrodes and the functional layer, which further affects the endurance and data retention of the device. As a result, SRMs based on conductive filaments or continuous conductive pathways tend to be significantly less reliable than SRMs based on electromigration. However, the oxide films underlying SRMs based on electromigration to achieve high RR, NL, on/off ratio, and fast switching are as thin as possible to 10 nm or even less than 5 nm, which may be subject to localized breakdowns and thus lead to SRM failures. Meanwhile, the continuous conductive pathways are the basis for ensuring a large on/off ratio [31, 40], creating a significant contradiction.

**Table 1:** Comparison of all key parameters of advanced SRMs

Structure	Year	RR	NL	On/off Ratio	Leakage Current	Operating Voltage	Speed	Retention	Endurance	Scalability	Applications for beyond CMOS	CMOS Compatibility	Refs
Ta/HfO_x_/ZrO_y_/Pt	2025	10^4^	7 × 10^3^	8 × 10^4^	< 1 pA	2.5 V	2 μs	10^4^ s	10^4^	–	–	Yes	[60]
Ag/CZTSSe/Mo	2025	1.5 × 10^3^	-	1.5 × 10^3^	> 10 μA	0.3 V	–	–	150	–	Security	No	[80]
Pt/TaO_x_/TiN	2025	10^7^	10^7^	-	< 1 nA	0.8 V	0.5 ms	–	–	–	RC	Yes	[81]
Pt/HfO_2_/Ta_2_O_5−x_/Ti	2025	10^4^	10^4^	10^3^	< 10 pA	3 V	0.5 ms	10^4^ s	10^5^	96 Kb	Flexible	Yes	[82]
Pt/HfO_2_/TiN	2025	10^8^	10^5^	10	< 0.1 pA	1.5 V	1 ms	–	10^7^	25.4 Tb	ADS	Yes	[51]
TiN/ANTO/TiN	2025	10^4^	10^4^	100	< 1 pA	1 V	10 ms	10^4^ s	3.5 × 10^4^	–	ANN	No	[83]
Ag/P(VDF-TrFE):BA 2PbI4/ITO	2025	10^6^	10^6^	10^6^	< 10 pA	0.1 V	–	10^4^ s	200	–	–	No	[66]
Pt/HfO_2_/Ti	2025	10^5^	10^3^	10^3^	< 10 pA	1.5 V	–	10^3^ s	100	8.9 Mb	CNN	Yes	[84]
Pd/TiO_2_/Ti	2025	7 × 10^6^	–	100	< 1 pA	3 V	1 μs	–	2 × 10^4^	–	Self-supervised learning	Yes	[46]
Pt/WO_3_/WO_3-x_/TiN	2024	10^5^	10^5^	10^3^	< 1 nA	2.5 V	–	10^6^ s	10^4^	180.3 Gb	ANN	Yes	[31]
Pt/Bi_2_O_2_Se/β-Bi_2_SeO_5_/Au	2024	10^5^	10^5^	10^5^	< 10 pA	3 V	20 ns	–	–	–	–	No	[40]
Pt/HfO_2_/WO_3-x_/TiN	2024	10^6^	10^5^	–	< 1 pA	2 V	0.5 ms	5 × 10^4^ s	10^6^	21 Gb	ANN	Yes	[29]
Pt/ZnO/WO_3-x_/Ta	2024	10^6^	100	10	< 10 pA	2 V	1 ms	10^6^	10^3^	–	RC	Yes	[30]
Pt/HfO_2_/CuTeHO	2024	10^5^	–	10^5^	< 1 pA	5 V	0.3 ms	10^3^ s (100 ℃)	10^6^	–	Hardware security	No	[47]
Au/FeO_x_/HfO_x_/Ag	2024	10^3^	–	10^4^	< 1 μA	2 V	50 ns	–	10^4^	–	CNN	No	[85]
Ru/NbO_x_/TiO_2_/TiN (16 layers)	2024	10^7^	10^5^	–	< 10 pA	2 V	600 ps	10^4^ s	10^10^	4 Tb	ANN	No	[49]
Pt/TiO_2_/NiO/ITO	2024	10^3^	10^4^	–	< 1 nA	3 V	–	10^3^ s	–	–	Optoelectronic ANN	Yes	[86]
TiN/TiO_2_/NbO_x_/NiO/Ru	2024	10^5^	10^4^	–	< 0.1 nA	2 V	–	–	–	1 Tb	Optoelectronic RC	No	[37]
Au/TiO_2_: Na,Al/Au	2024	10^4^	–	100	< 0.1 nA	2 V	5 μs	7 × 10^4^ s (125 ℃)	5 × 10^5^	–	ANN	No	[61]
Au/ Perovskite/ITO	2024	514	–	10^3^	< 10 nA	3 V	–	10^4^ s (85 ℃)	10^4^	3 Mb	–	No	[87]
Pt/NbO_x_/Ti	2024	100	10	10	< 0.1 mA	4 V	1 ms	–	10^3^	–	RC	No	[88]
Pt/Al_2_O_3_//HfO_x_/TiO_x_/Al	2024	10	–	–	< 10 μA	3 V	1 μs	–	10^3^	1 Kb	RC	Yes	[89]
Ru/Hf_0.8_Si_0.2_O_2_/Al_2_O_3_/Hf_0.5_Si_0.5_O_2_/TiN	2024	10^3^	10^2^	–	< 10 pA	3 V	100 μs	10^4^ s (85 ℃)	10^6^	–	ANN	Yes	[32]
Au/TiO_x_/FTO	2024	100	10^3^	–	< 10 μA	2 V	–	–	–	–	Optoelectronic RC	No	[90]
Pt/WO_x_/W (3 layers)	2024	4.8 × 10^2^	–	4.1 × 10^2^	< 1 nA	1.5 V	0.5 ms	–	10^5^	–	RC	Yes	[45]
Pt/TiO_2-x_: F/Ti	2024	10^5^	–	10^4^	< 0.1 nA	3 V	77 ns	–	10^6^	–	–	No	[42]
Pt/NiO_x_/ WO_3−x_: Ti/W	2023	10^2^	100	10	< 0.1 nA	3 V	500 ms	–	500	–	SOM	Yes	[33]
Pt/HfO_2_/TaO_x_/Ta (2 layers)	2023	10^4^	10^4^	10	< 10 pA	5 V	200 ns	10^4^ s (85 ℃)	10^5^	1.56 Gb	–	Yes	[91]
Pt/Ta_2_O_5_/ HfO_2_: Al/TiN (2 layers)	2023	10^4^	10^4^	10^3^	< 10 pA	10 V	–	100 s	100	–	Logic	Yes	[92]
Pt/HfO_2_/TaO_x_/Ta	2023	10^4^	10^4^	10^3^	< 0.1 pA	6 V	1 μs	3 × 10^4^ s (125 ℃)	10^6^	95 Mb	Sparse matrix multiplication	Yes	[17]
Pt/Ta_2_O_5_/Nb_2_O_5-x_/Al_2_O_3-y_/Ti	2023	5 × 10^4^	–	10^3^	< 0.1 nA	10 V	10 ms	2 × 10^5^ s (150 ℃)	10^5^	–	ANN	No	[93]
Pt/Al_2_O_3_/HfO_2_/TiN	2023	10^6^	10^3^	–	< 1 pA	3.5 V	–	10^3^ s	10^6^	–	Graph analysis	Yes	[35]
TiN/ TiO_x_N_y_/TiO_x_/NbO_x_/Ru	2023	10^6^	5 × 10^3^	–	< 1 pA	2.5 V	200 ns	10^4^ s (125 ℃)	10^8^	10 Gb	–	No	[53]
Ru/HfZrO/TiN	2022	100	100	10	< 10 pA	3.5 V	1 μs	–	–	–	RC	Yes	[94]
Pt/Al_2_O_3_/TaO_x_/Ta	2022	10^4^	10^4^	10^3^	< 0.1 pA	10 V	20 ms	10^4^ s (125 ℃)	10^4^	538 Mb	–	Yes	[70]
Pt/TiO_2_: Na/Pt	2022	10^4^	10^4^	–	< 0.1 nA	10 V	5 ms	10^5^ s	10^4^	–	CNN	No	[36]
Pt/HfO_2_/Nb_2_O_5_/HfO_2_/Ti	2022	10^5^	–	–	< 10 pA	7 V	–	10^3^ s	10^4^	–	Stashing	No	[63]
Au/TaO_x_/Al_2_O_3_/TiN	2021	73	236	100	< 1 nA	3 V	80 μs	2.5 × 10^3^ s	100	160 Kb	–	Yes	[64]
Pt/TaO_x_/Ta	2021	10^5^	10^5^	10^4^	< 10 pA	14 V	–	10^4^ s (85 ℃)	10^3^	160 Mb	Logic	Yes	[57]
Au/h-BN/graphene/h-BN/Ag	2019	10^10^	–	10^3^	< 10 fA	5 V	50 ns	10^6^ s	10^6^	1 Tb	MVM	No	[41]
TiN/HfO_2_/TaO_x_/Ti/TiN/W (8 layers)	2017	100	100	100	< 10 pA	6 V	300 ns	10^4^ s (125 ℃)	10^7^	–	–	Yes	[95]
Pt/Ta_2_O_5_/ HfO_2_/TiN	2016	2 × 10^3^	–	–	< 1 pA	15 V	–	10^4^ s (125 ℃)	10^3^	–	–	Yes	[39]
Pt/NbO_x_/TiO_y_/NbO_x_/TiN	2016	10^5^	10	–	< 1 pA	12 V	-	3 × 10^3^ s	10^4^	1 Mb	–	No	[43]
Ru/HfO_2_/TiO_x_/TiN	2016	10^3^	10^3^	–	< 0.1 pA	3 V	400 ns	10^4^ s	10^7^	10 Mb	–	Yes	[96]
TiN/ HfO_2_/HfO_2_: Si/TiN	2016	300	300	10	< 1 pA	5 V	–	10^8^ s	–	–	–	Yes	[97]
Pt/Ta_2_O_5_/HfO_2−x_/Ti	2015	10^6^	–	10^6^	< 1 pA	8 V	–	10^4^ s (200 ℃)	–	–	–	Yes	[44]
TiN/HfO_2_/CuGeS/W	2015	10^3^	10^3^	10^3^	< 0.1 pA	4 V	1 μs	10^4^ s	10^7^	10 Mb	–	No	[98]

With RR and NL increasing, the increased barrier height may lead to more significant stress on the device materials during repeated switching cycles, potentially reducing the device’s lifespan [72]. High barrier height values can also sometimes be achieved at the expense of data retention. The increased barrier heights and reduced current flow can lead to slower relaxation processes, potentially causing the device to switch back to the off-state over time. This is particularly problematic in applications requiring long-term data storage. Additionally, the higher operating voltages required to overcome these barriers can exacerbate Joule heating effects, further degrading device performance over time [12, 51]. Lowering the operating voltage to increase switching speed can reduce the stress on the device materials [73], potentially improving endurance. However, if the operating voltage is too low, it may not be sufficient to drive the necessary switching processes, leading to incomplete state transitions and reduced device reliability [74]. To balance the trade-offs of RR (or NL), speed, and endurance, one optimal approach is to further optimize the device structure and materials to achieve a moderate RR or NL while maintaining acceptable switching speed and endurance. For instance, using thin insulating layers and optimizing the doping levels can help reduce the energy barriers without significantly compromising RR and NL [42], and advanced materials with high thermal stability and low defect densities can improve endurance while maintaining high RR and NL. Moreover, using materials with high ionic mobility and optimizing the device dimensions can help achieve faster switching without significantly increasing power consumption [75, 76]. As an example, Tan et al. introduce a self-rectifying two-dimensional memtransistor, employing asymmetric metal contacts—a Schottky Platinum contact and a quasi-ohmic Bismuth contact and integrating memristor resistive switching with transistor gate tunability for advanced neuromorphic computing [77].

From the perspective of the fabrication process, the fabrication of SRMs involves several critical steps, including material deposition, annealing, and doping, each of which can significantly influence the device’s performance. Understanding how specific process variations affect key performance metrics is also essential for developing more reliable and consistent SRM fabrication processes. First, the thickness of the active layer in SRMs is a critical parameter that affects both the RR and NL. Thicker layers generally enhance the RR and NL by increasing the energy barriers under enough forward bias [29]. Second, annealing temperature plays a crucial role in determining the crystallinity and defect density of the active layer. Higher annealing temperatures can improve the crystallinity, leading to lower defect densities and enhanced device performance [78]. However, excessively high temperatures can cause material degradation or unwanted phase transitions, negatively impacting the device's stability and performance. Last but not least, doping is a common technique used to control the electrical properties of the active layer in SRMs [42]. The concentration of dopants can significantly affect the device's on/off ratio, switching speed, and endurance. Moderate doping concentrations can increase the conductivity of the active layer, enhancing the on/off ratio and switching speed. Recently, Wang et al. epitaxially grew the AlScN film on a silicon substrate for reliable SRMs, whose crystallinity, surface roughness, and ferroelectric properties were meticulously optimized via dual-target nitrogen reactive magnetron sputtering, fine-tuning the doping ratio [79].

In summary, balancing all the metrics of SRMs while ensuring superior reliability is difficult to achieve. And current state-of-the-art SRMs are still operated at higher voltages (> 1.5 V) [17, 37, 46]. Possible strategies include using multilayer stacking for fine control of the conductive pathways, optimizing the precision of the CMOS process, introducing isolation layers around the device, and protecting the device using encapsulation processes, etc. The next phase of exploration will be based on the CMOS process, the matching of the available functional materials, the high temperature reliability characterization with lower and lower operating voltage to ensure that the reliability is as synergistic as possible with the optimization of the key metrics. Besides, optimizing the performance of SRMs involves carefully balancing multiple key metrics. By understanding the trade-offs between these metrics and tailoring the device design to specific application requirements, SRMs can be optimized for high performance, reliability, and scalability in various beyond-CMOS computing paradigms. It is worth noting that all key terms mentioned in this paper are summarized and explained in Table 2.

**Table 2:** Key glossary

Term	Abbreviation	Definition
Self-rectifying memristor	SRM	A novel type of memristor that exhibits intrinsic diode-like rectification, enabling unidirectional conduction and suppressing sneak path currents in crossbar arrays
Rectification ratio	RR	The ratio of the on-state current of the device under positive bias to the off-state current under negative bias, indicating the level of current suppression in reverse bias
Nonlinearity	NL	The ratio of the current of the device at the read voltage under the low-resistance state (LRS) to its current at the partial read voltage, indicating the degree of nonlinearity in the current–voltage characteristics
Complementary metal–oxide–semiconductor	CMOS	A widely used technology for manufacturing integrated circuits, characterized by low-power consumption and high scalability
Vector–matrix multiplication	VMM	A fundamental operation in many computing tasks, where a vector is multiplied by a matrix to produce a result vector, often used in neural networks and in-memory computing
Ternary content-addressable memory	TCAM	A type of cell that allows data to be retrieved based on its content rather than its address, often used in high-speed search applications
Artificial neural network	ANN	A computational model inspired by the structure and function of biological neural networks, used for tasks such as image recognition and pattern classification
Convolutional neural network	CNN	A type of neural network that uses convolutional layers to process data with grid-like topology, commonly used for image and video recognition tasks
Autonomous driving systems	ADS	Systems that enable vehicles to operate without human intervention, often using advanced sensors, computing, and machine learning techniques
Reservoir computing	RC	A type of recurrent neural network that uses a fixed, highly dynamic reservoir to map input signals to a high-dimensional space, followed by a linear readout layer for output
Physical unclonable function	PUF	A security primitive that generates unique and unclonable digital fingerprints based on the inherent physical variations in a device, used for authentication and key storage
True random number generator	TRNG	A hardware device that generates random numbers based on physical processes, providing high entropy for cryptographic applications
Homomorphic encryption	HE	A form of encryption that allows computations to be performed on ciphertext, producing an encrypted result that, when decrypted, matches the result of operations performed on the plaintext

## Applications of SRM in Beyond CMOS

In the previous section, we provided a detailed discussion of the working principle, conductive mechanism, and unique features of SRMs. Based on these, SRMs are capable of a large number of cutting-edge applications for beyond CMOS. The great scalability potential of SRMs provides a solid hardware foundation for ultra-high-precision in-memory computing [99], neuromorphic computing [50], and hardware security [100].

### In-Memory Computing

SRM-based in-memory computing utilizes the non-volatile, high-density, and programmable characteristics of memristors to efficiently perform vector matrix multiplication (VMM) by reading the rows and collecting current along the columns of memory cells, thus realizing the deep integration of memory and computing [101].

#### Regular VMM

For regular VMM, the memristors are distributed as storage units at the intersection of word lines (WLs) and bit lines (BLs), and the writing and updating of the memristor resistance state can be realized by controlling the voltage of WLs, while BLs are used to read the current signals of the memristors to obtain the stored data. By storing the weights of the matrix in the conductance values of the memristors and applying the voltage signals of the input vectors on the word lines, the current of each memristor is proportional to its conductance value according to Ohm’s law. The bit line collects the currents of all the memristors through Kirchhoff’s law, thus directly outputting the result of the VMM [102].

As mentioned earlier, SRMs can effectively suppress leakage currents in passive crossbar arrays, thereby improving read accuracy and data accuracy. Further, the SRM cell-based crossbar arrays are able to perform multiply-accumulate computation (MAC) in a massively parallel manner. This parallelism allows the computational complexity of the VMM to be reduced from the traditional *O*(*n*^2^) to *O*(*n*) or even better [103, 104], significantly improving the computational efficiency. In recent years, a large number of state-of-the-art SRMs with applications to regular VMM have been developed. For example, Zhao et al. developed an SRM based on a quasi-free-state Bi_2_O_2_Se single-crystal thin film, achieving fast switching (< 20 ns) and low-power consumption (< 1.2 pJ) [40]. In 2019, Sun et al. introduced an SRM based on a van der Waals heterostructure of hexagonal boron nitride (h-BN) and graphene, achieving self-selectivity in excess of 10^10^, switching ratios in excess of 10^3^, and terabit-level scalability [41].

**Fig. 5:**
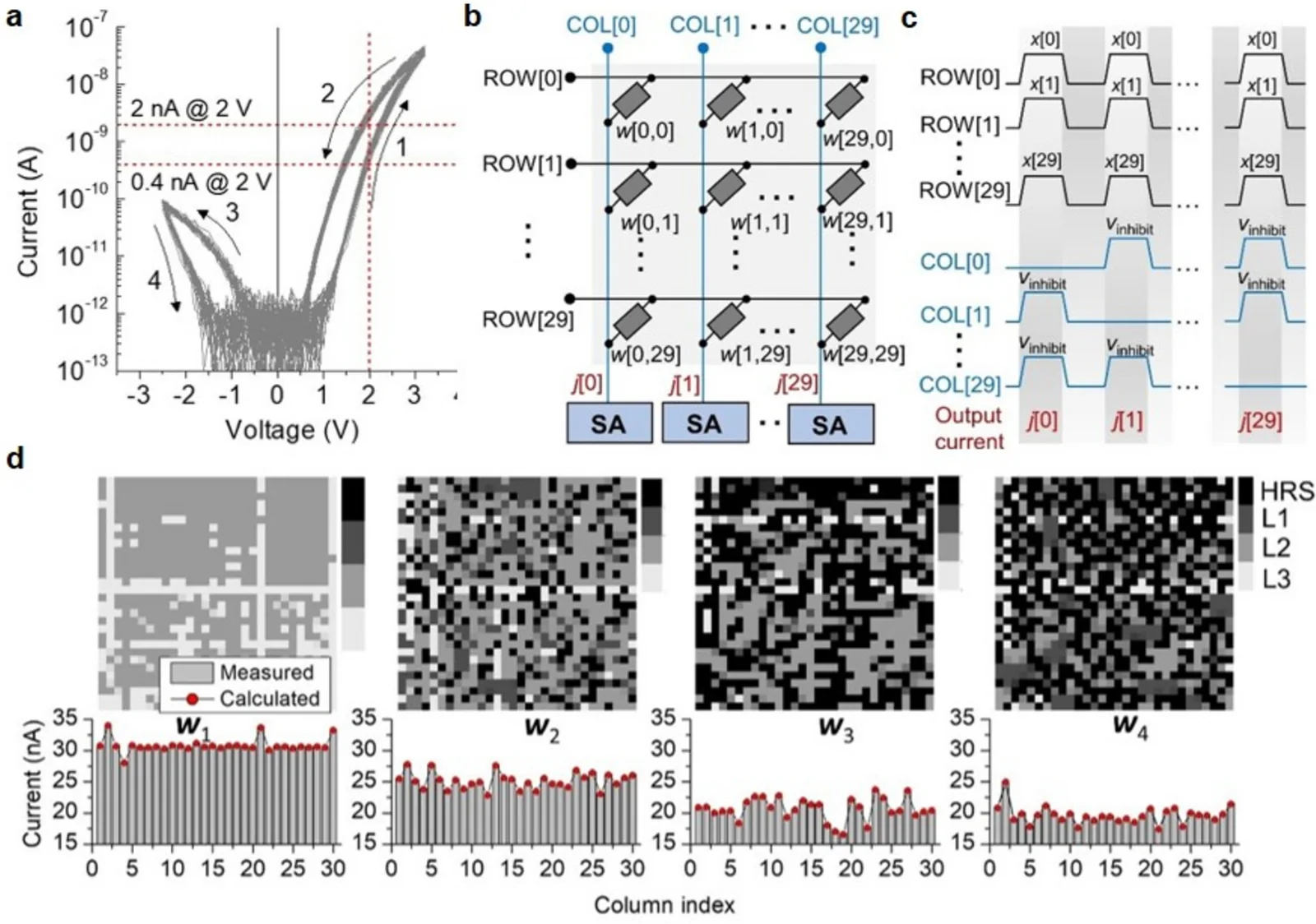
Passive crossbar arrays based on SRMs for regular VMM. **a** DC I–V curves of SRMs based on Ru/Hf_0.8_Si_0.2_O_2_ /Al_2_O_3_/Hf_0.5_Si_0.5_O_2_/TiN structures, serving as cells of passive crossbar arrays. **b** Scheme of 30 × 30 matrix mapped onto a crossbar array of the same size. **c** Schematic timing diagrams of the row and column line signals, with inhibit voltages applied to unchosen column-lines. **d** Conductance maps and measured current vectors of four random matrices (*w*_1_–*w*_4_). Reproduced from [34], with permission from Springer Nature

In 2021, SRMs based on Ru/Hf_0.8_Si_0.2_O_2_ /Al_2_O_3_/Hf_0.5_Si_0.5_O_2_/TiN structures, with DC I–V curves shown in Fig. 5a, were used to construct 30 × 30 passive crossbar arrays [34]. The group selected four random matrices with different sparsities in the experiment and mapped them onto the passive crossbar array. The efficiency and accuracy of the arrays in handling large-scale matrix operations were verified by quantizing the currents using sense amplifiers at the end of the column lines (Figs. 5b and 3c). The experimental results show that the measured current vectors are almost identical to those obtained by extrapolating the currents from individual cells, indicating that the interference of unselected cells is negligible even in large-scale arrays (Fig. 5d). At the same time, the power consumption level of the realized VMM is much lower than that of conventional computing architectures, especially when dealing with intensive matrix operations. This suggests that SRM-based crossbar arrays are not only advantageous in terms of computational efficiency, but also show great potential in terms of energy efficiency. In addition, related concerns were reasonably presented. Despite the excellent performance of crossbar arrays in in-memory computation, there are still some challenges to achieve the desired computational temporal complexity *O*(1). Theoretically, by activating all column lines simultaneously, the crossed-bar array enables on-the-fly computation of VMM. However, in practice, the finite line resistance leads to uneven cell voltage distribution, which affects the computational accuracy. In addition, activating all column lines simultaneously requires separate sense amplifiers and subsequent logic circuits for each column line, which may lead to additional area overhead in large-scale arrays.

With the unique property of SRMs to significantly suppress the sneak path currents in passive crossbar arrays, the realization of a massively integrated VMM can significantly enhance the parallel processing capability of the hardware while significantly reducing the computational temporal complexity.

Compared to single-layer SRMs, 3D-stacked SRMs have significant advantages for in-memory computing integration [105, 106]. First, the 3D-stacked structure can fully utilize the vertical space and significantly increase the storage density and computing power per unit area. This high-density integration not only increases storage capacity, but also significantly enhances the computing power of the in-memory computing chip, making it more suitable for processing large-scale data and complex computing tasks [12]. What’s more, the 3D-stacked memristors can be seamlessly integrated into existing CMOS processes [107]. In the recent decade, there has been little research on in-memory computing with 3D SRMs, but the related device mechanism has become much hotter [53, 91, 95, 96, 98]*.*

#### Content Addressing

Content addressing is a method for organizing and retrieving data based on the data’s intrinsic content, rather than its storage location [108]. In this approach, each data object generates a unique identifier through its content. SRM arrays achieve content addressing through their unique in-memory computing function, which utilizes self-rectifying features to suppress the sneak path current, thus ensuring accurate data storage and efficient retrieval [109, 110]. Fast content-based access and processing is achieved by mapping the hash value of the data content to specific SRM cells and performing logical operations directly on these cells. Among them, three-state content-addressable memory (TCAM) supports three states of “0,” “1” and “don't care” in each SRM cell with flexible matching operations realized through masks [111].

**Fig. 6:**
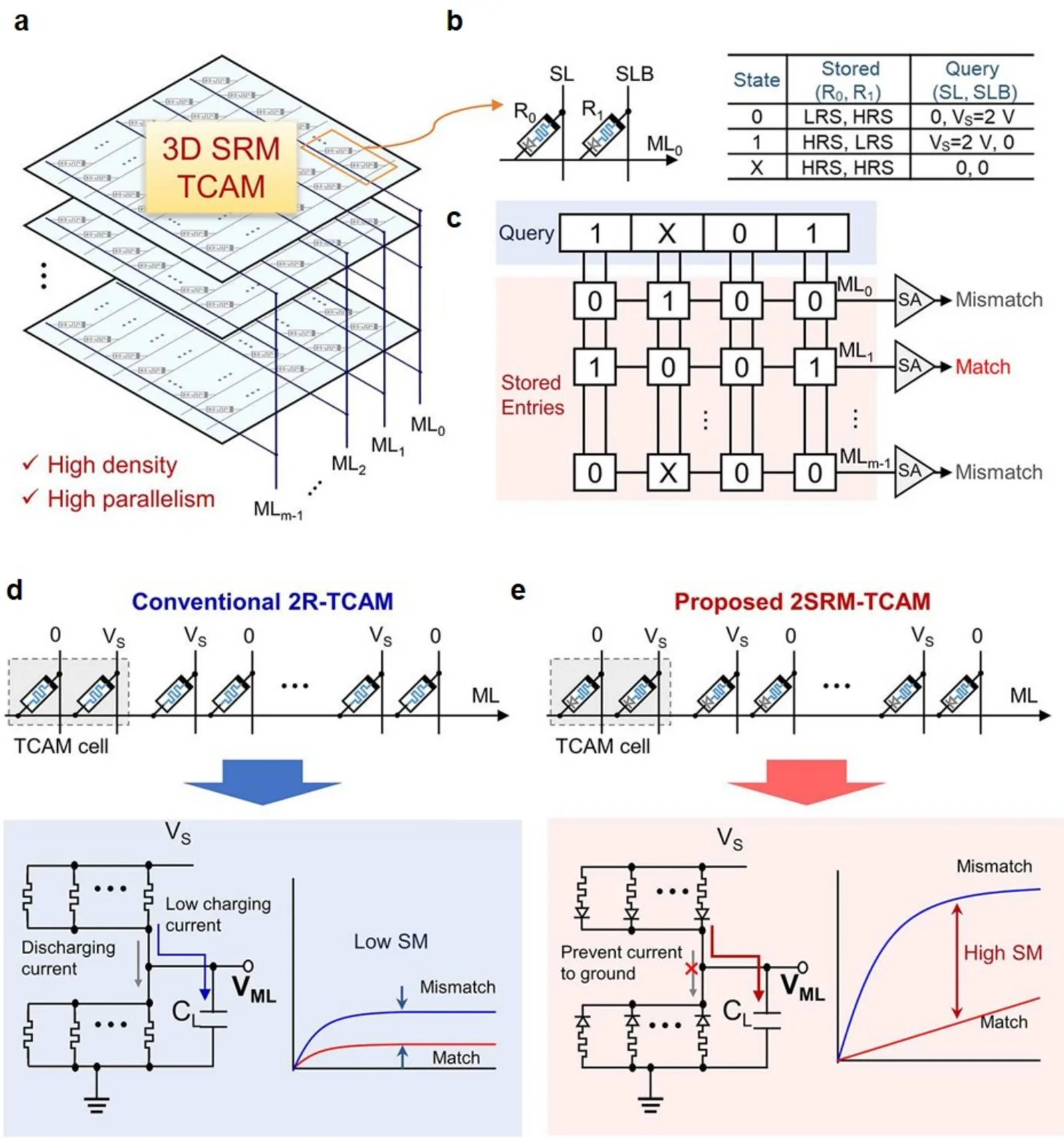
TCAM based on SRMs. **a** Schematic configuration of the 3D SRM-based TCAM array. **b** Cell structure and state definitions of a 1-bit SRM-based TCAM. **c** Schematic diagram of SRM-TCAM arrays for performing the exact match. ML charging circuit models for **d** 2R-TCAM and **e** SRM-TCAM. Reprinted from [48], Copyright 2025 Springer Nature

In 2018, Chen et al. developed a Ge-based SRM using a full CMOS-compatible technology and a thin AlO_x_/GeO_x_ interfacial layer, demonstrating excellent switching and rectification properties [109]. Based on this device, they propose and validate a high-density nonvolatile TCAM whose functionality is verified by experimental measurements. Wang et al. experimentally verified the parallel search function of a 2-bit TCAM array based on a Ge-based SRM [112]. With a sub-nanosecond ultrafast measurement system, it is confirmed that the search energy consumption of this TCAM is as low as 1.0 fJ/bit/mismatch, and the search operation can be completed within 200 ps, which significantly improves the data retrieval speed. The match reliability of Ge-based SRM-based TCAM cells combined with their full CMOS compatibility validates their potential for scaling up to ultra-large-scale TCAM systems. Moreover, the outstanding advantages of SRM suppression of sneak path currents applied to TCAM cell design were particularly emphasized by Goh et al. [110]. By employing a TiN/HZO/TaN/W stacked structure, the realized ferroelectric tunnel junction (FTJ) exhibits a rectification ratio of up to 1000 and a tunneling electroresistance of about 100, preliminarily demonstrating its characteristic of preventing crosstalk between array cells. This FTJ-based TCAM cell structure achieves a compact area (only two FTJ cells are required) and exhibits high endurance (10^8^ cycles) and low-power consumption, while achieving about 90% accuracy in pattern recognition tasks, providing a highly promising solution for high-density, low-power TCAM applications [110]. This approach markedly enhances the density and reliability of the TCAM while simultaneously decreasing power consumption and error rate. Yu et al. [48] introduced a 3D SRM-TCAM that significantly advanced in-memory search capabilities. As illustrated in the schematic (Fig. 6a), the memory array adopts a compact 3D vertical architecture, where multiple storage layers are stacked to achieve high integration density and N-fold improvement in search parallelism. The fundamental building block is a novel TCAM cell (Fig. 6b) consisting of just two SRMs connected to a common match line (ML), storing ternary states through different resistance state combinations of the two devices. This minimalist design enables efficient implementation within a 3D crossbar array for parallel exact matching operations (Fig. 6c). The crucial advantage emerges when comparing the operational mechanisms with conventional designs. While the traditional two-memristor TCAM (Fig. 6d) suffers from insufficient ML charging due to simultaneous charging and discharging paths, the SRM-based counterpart (Fig. 6e) benefits from the self-rectifying characteristic that functionally creates a one-diode–one-resistor structure. This effectively blocks the discharge path to ground, allowing more adequate charging current and consequently a significantly larger sense margin.

Obviously, in the development of SRM applications for content addressing, researchers have focused on the CMOS compatibility of the selected materials to confirm the feasibility of the proposed TCAM architecture for large-scale scaling [113]. Nonetheless, the SRM devices utilized in the TCAM cells at this stage are still unable to achieve substantial RR and NL, which fundamentally prevents excellent scalability.

#### Other Applications

In the field of in-memory computing based on SRMs, except regular VMM and content addressing, there are unique applications being initially developed [17, 47, 57, 92].

First, for basic logic functions, Ni et al. [57] verified that controlled majority-inverter graph logic based on SRMs offers significant advantages in terms of computational complexity, enabling the implementation of complex logic functions, such as 1-bit full adder and 4-bit square root computation, with fewer devices and steps. In addition, parallel logic gates based on SRMs are capable of implementing priority encoders and XOR logic functions through logic cascading [92].

**Fig. 7:**
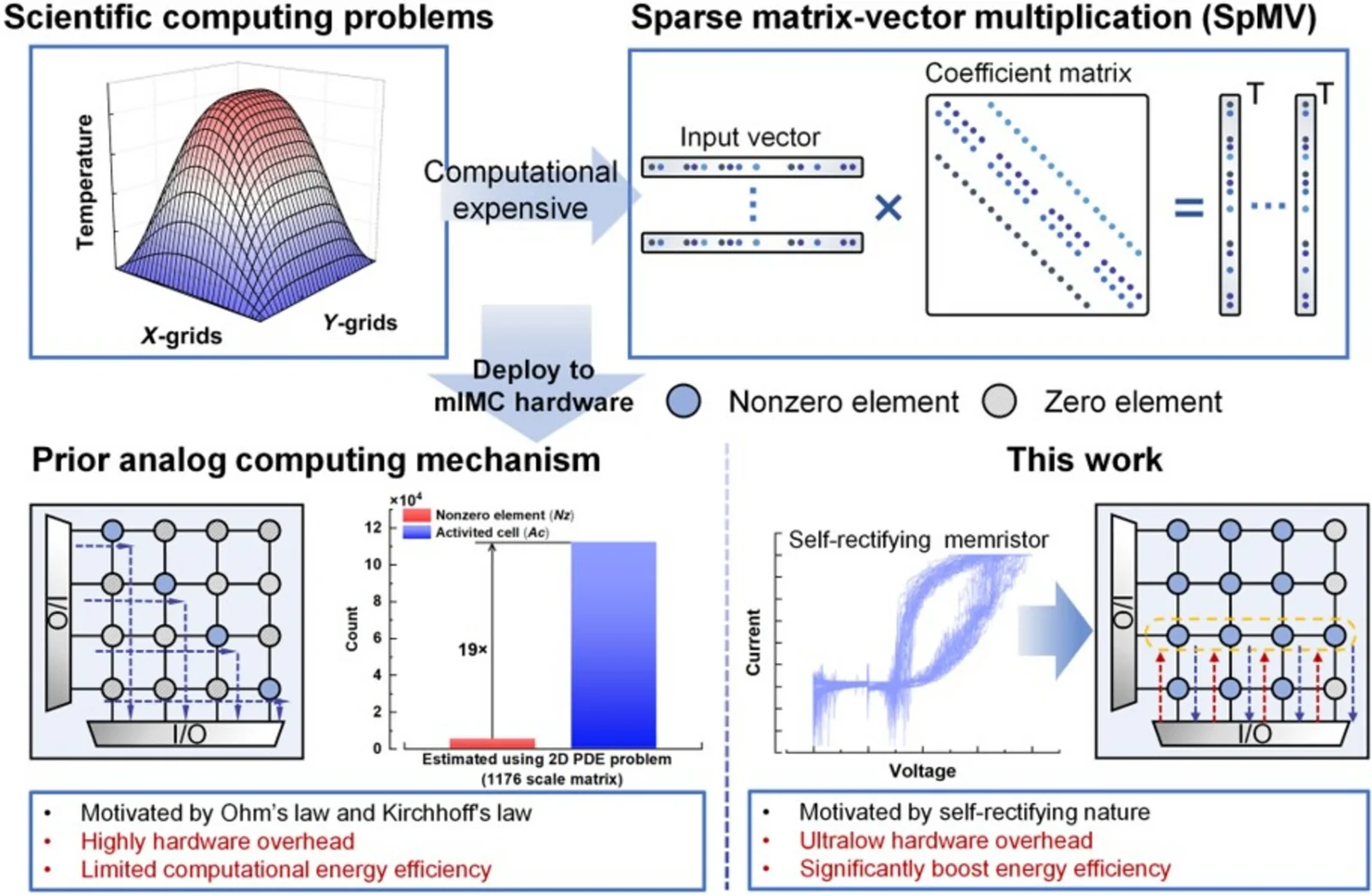
Motivation and metrics of the proposed sparse matrix multiplication based on SRMs. Reprinted from [17], Copyright 2023 American Association for the Advancement of Science

Besides, in many in-memory computating scenarios, most of the elements in the matrix are zero, while the nonzero elements account for only a few. This makes traditional matrix multiplication methods inefficient when dealing with sparse matrices, as a large amount of computational resources are wasted on operations with zero elements. Sparse matrix multiplication significantly reduces computational complexity and storage requirements by optimizing storage and computation methods to operate only on nonzero elements [114]. SRMs are very suitable for sparse matrix multiplication due to their powerful self-rectification capability. In 2023, Li et al. significantly reduced the energy consumption and hardware overhead at the hardware level by compressing the storage format of sparse matrices, mapping nonzero elements into memory arrays, and utilizing the low-power and rectification characteristics of SRMs with both of RR and NL exceeding 10^4^ and ultra-low leakage current below 0.1 pA to suppress crosstalk currents (Fig. 7) [17]. Experimental results show that the system achieves a performance of about 97 to 11 TOPS/W in 2- to 8-bit sparse computation tasks, which improves the energy efficiency by more than 85 times and reduces the hardware overhead by about 340 times compared to conventional memory computation systems.

With its non-volatile, high-density and self-rectifying features, SRM demonstrates high-performance and low-power consumption advantages in in-memory computing paradigm, significantly enhancing computational accuracy and parallel processing capability by suppressing leakage current. In addition, SRMs support a 3D-stacked structure, which further enhances the density and performance of in-memory computing, and are compatible with CMOS processes, making large-scale applications possible. In special applications such as content addressing and sparse matrix multiplication, SRM further reduces hardware overhead and power consumption by optimizing the storage format and reducing invalid calculations, demonstrating the potential for a wide range of applications with much more mature CMOS compatibility.

### Neuromorphic Computing

Relying on in-memory computing, neuromorphic computing is a brain-inspired computing paradigm that aims to achieve efficient information processing and learning capabilities by modeling the structure and function of biological neural networks [115], owning dominant status in the beyond-CMOS computing paradigm. It achieves brain-like parallel computing and learning functions by constructing brain-like networks of neurons and synapses simulating synaptic weight changes. Although You et al. present a significant advancement by developing a dynamic SRM that integrates leaky integrate-and-fire neuron emulation and refractory period simulation [116], applications of SRM-based neuromorphic computing have primarily focused on emulating synaptic behaviors currently. Compared with traditional computing architectures, the advantages of neuromorphic computing lie in its high degree of parallelism and efficient processing of complex tasks, while its uniqueness lies in its ability to mimic the plasticity and dynamic behaviors of the brain, such as short- and long-term synaptic plasticity, as well as learning through mechanisms such as spiking timing-dependent plasticity (STDP) [115, 117]. A prerequisite for embedding into a neuromorphic computing system is that the selected SRMs should have sufficient dynamic properties [42]. Moreover, SRMs can provide a massively scalable hardware foundation for existing mature neuromorphic computing architectures, further improving computational efficiency and energy efficiency ratio.

#### Artificial Neural Network

The computational accuracy of traditional artificial neural networks (ANNs) is limited by arithmetic power when running on CPUs or GPUs. When dealing with large-scale image recognition tasks, memristor-based hardware can be more easily scaled to larger network structures, thus breaking through the bottleneck of the traditional algorithm ANN that is difficult to scale with limited hardware resources [118]. Further, dynamic SRMs could provide synaptic characteristics with great scalability for high-precision hardware-based ANN.

**Fig. 8:**
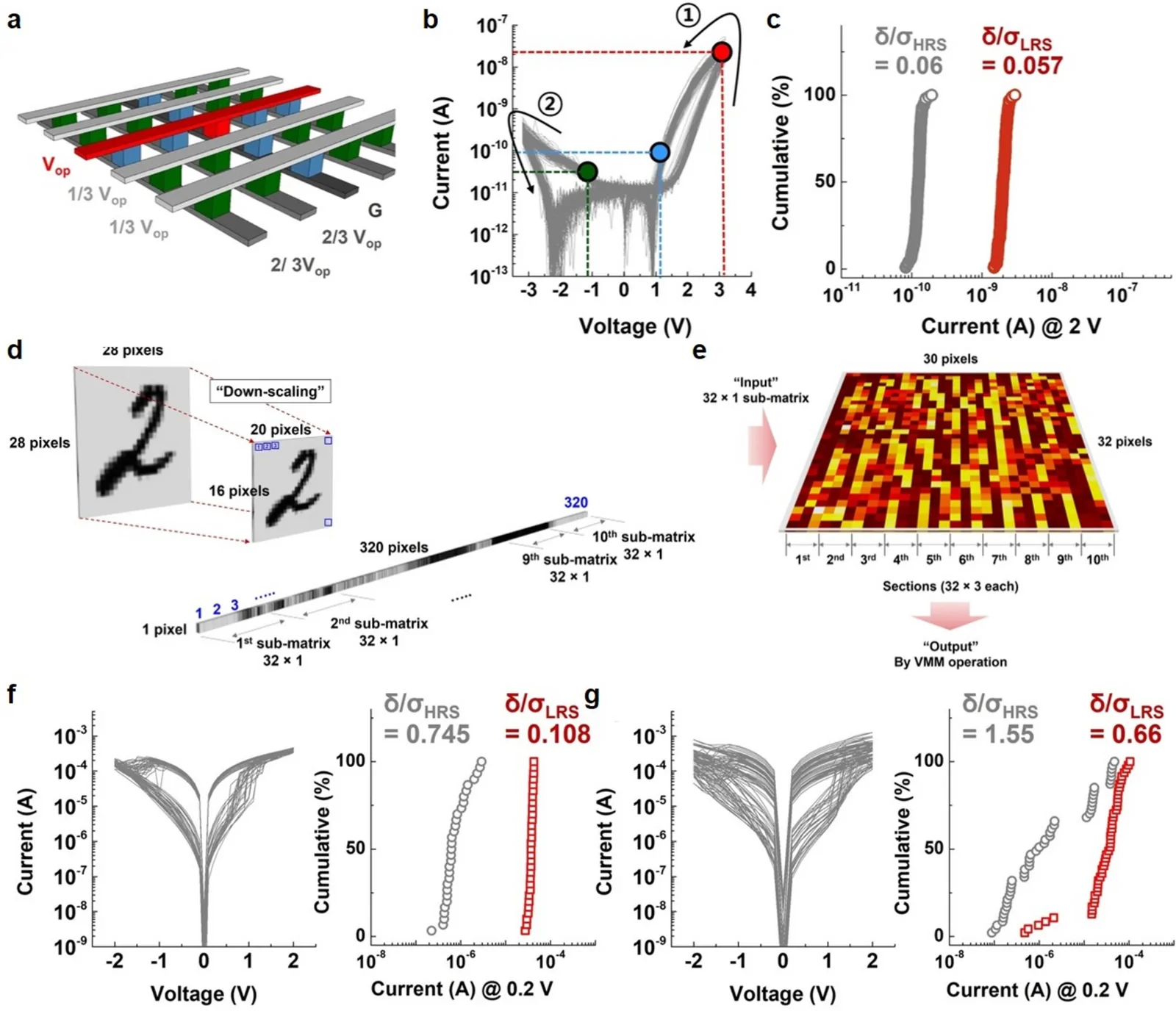
A novel ANN accelerator based on 1-kb SRM array. **a** Schematic diagram of 1/3 bias scheme of the passive crossbar array. **b** DC I–V curves of HfSiO_x_-based SRMs. **c** Cumulative probability of resistance states in the 1-kb crossbar array with low device-to-device variation less than 6%. **d** Schematic diagram of downscaling. **e** Schematic diagram of VMM operation based on 1-kb passive crossbar array. **f** DC I–V curves of one memristor without rectification (left). The cumulative probability distribution of the read current where each state is extracted from the DC I–V curves (right). **g** DC I–V curves (left) of memristors in an 8 × 8 passive crossbar array. The cumulative probability distribution of the read current for each state is extracted from the DC I–V curves (right). Reproduced from [32], with permission from Springer Nature

In 2018, the first SRMs-based ANN was constructed via nanoporous TaO_x_-based SRMs, and the device exhibited high nonlinearity, low synaptic coupling, good endurance, and excellent retention stability [62]. The synaptic device successfully modeled the key functions of long-term potentiation (LTP), long-term depression (LTD), and STDP and achieved a high accuracy of 89.08% in MNIST image recognition with only 15 training cycles. This work confirms the great potential of SRMs in developing neuromorphic computing, provides a promising synaptic device platform for building high-density, low-power ANNs with high learning capabilities and provides initial guidance for related work in the following years [63, 93, 119]. More researches related to how SRMs can be more deeply integrated into ANN and the all-hardware implementation of ANN has increased at the time of the outbreak from the beginning of 2024 [32, 49, 61]. Jeon et al. [32] explored in detail the application of SRMs in the construction of hardware accelerators for ANNs. They designed and prepared a 1-kb passive crossbar array that integrates HfSiO_x_-based SRMs, exhibiting high RR around 10^4^ (Fig. 8b), low device-to-device variation less than 6% (Fig. 8c), and excellent nonvolatility ensuring precise conductance adjustment. By adopting a 1/3 bias scheme (Fig. 8a), the SRMs can effectively suppress crosstalk currents from neighboring cells, ensuring the accuracy of VMM operation. This group utilized this 1-kb passive crossbar array for the MNIST handwritten digit classification task (Fig. 8d), and the weights obtained through software training were mapped to the conductance states of the array, achieving 100% classification accuracy (Fig. 8e). In addition, it was found that defective cells in the passive crossbar array significantly degraded the classification accuracy, whereas read margins had less impact on the classification task [32]. This suggests that SRMs with non-conducting filament mechanisms are ideal for ANN applications due to their high consistency and reliability. It is also quite noteworthy that they visualized the importance of the selection function in passive crossbar arrays through detailed comparative experiments for the first time, elucidating crossbar arrays lacking the selection function are unable to accurately perform the VMM operation, and thus fail to realize reliable ANN computation (Figs. 8f and 6g) [32]. Moreover, combining a record-breaking oversized RR of over 10^7^ and NL of 10^5^ with ultrafast response at the ps level provides another in-depth guide to the development of ANN hardware accelerators [49]. Besides, Kim et al. present an interface-type Al/N-doped TaO_x_ (ANTO) SRM engineered via ALD process to optimize oxygen vacancy concentration [83]. Hardware-level demonstrations based on the proposed doped SRMs confirm reliable multilevel programming, including conductance-mapped word patterns, highlighting its potential for high-density, energy-efficient neuromorphic computing.

For ANN, the weight updating linearity refers to the linear relationship between the weight changes and the input stimuli during the training process of the ANN. High linearity implies that the change of weights can more accurately reflect the change of external input stimuli, thus improving the learning efficiency and accuracy of ANN. Therefore, there are also corresponding researches focusing on the weight update linearity of ANN-oriented SRMs based on WO_3-x_ themselves, greatly compatible with CMOS processes [29, 33]. An unsupervised self-organizing mapping (SOM) neural network based on SRMs with vector coding and topological organization is highly resistant to noise and steep synaptic decay, breaking through the bottleneck of traditional ANN in classification accuracy [33]. Combined with the ultra-high weight-enhanced linearity of SRM itself, the recognition accuracy of the SOM network based on passive crossbar array can reach 98.41% after training 56 sets of samples, which is much higher than that of the traditional ANN in the direction recognition experiments [33].

#### Convolutional Neural Network

Convolutional neural networks (CNNs) need to perform a large number of sliding convolution operations when dealing with tasks such as image recognition. These operations involve a large number of MAC operations and thus require compute units that support parallel MAC to meet the associated arithmetic requirements [120]. Notably, SRMs have revolutionized the way all needs are met in a comprehensive manner aforementioned compared to conventional memristors.

Kim et al. prepared Na-doped SRMs with excellent performance using in situ doping by atomic layer deposition technique to achieve reliable reading and writing in 6 × 6 crossbar arrays [36]. On this basis, the group constructed a LeNet-5-based CNN for MNIST handwritten digit recognition simulation experiments. The network is trained in two stages, and the simulation process takes into account device non-idealities by quantizing the weights and adding noise. Ultimately, the Na-doped SRM crossbar array constructed CNN achieves a validation accuracy of 99.1% on the MNIST dataset, and the double-memristor scheme also achieves an accuracy of over 95% without pre-training [36]. Recently, the array based on Pt/HfO_2_/Ti SRMs, proposed by Zhao et al., successfully implements an 8-bit convolutional neural network in hardware, achieving 98% accuracy on MNIST handwritten digit recognition [84].

Despite preliminary research, hardware implementation of CNNs based on SRMs faces a number of challenges, including low yield and variation problems at the device level, computational inefficiencies due to the sequential nature of convolutional operations, and complex back-propagation and weight-shifting problems during the training process [120]. The related potential challenges are discussed in detail in the subsequent sections.

**Fig. 9:**
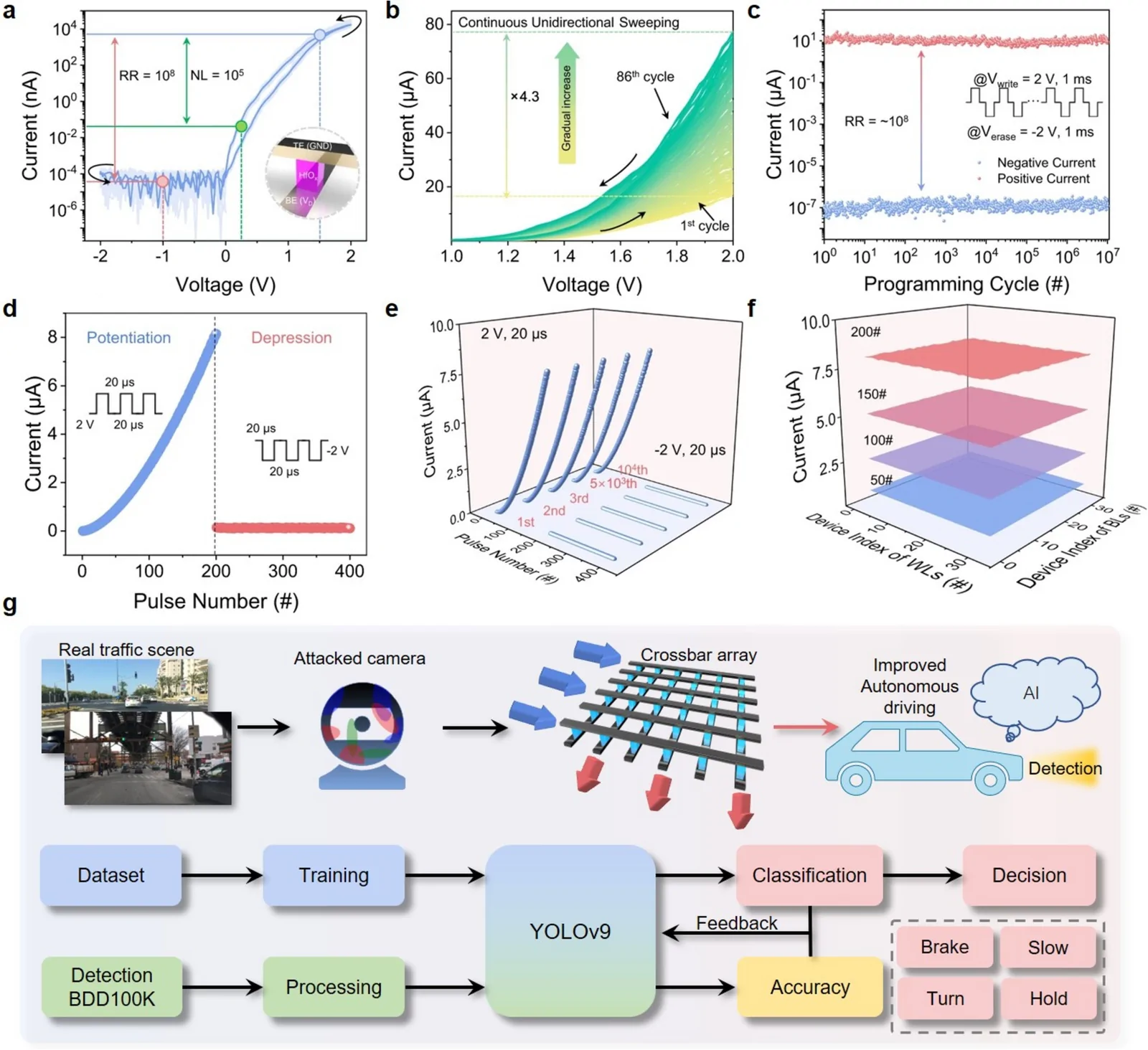
Hardware-level ADS system based on SRMs. **a** I–V characteristics of TiN/HfO_x_/Pt SRMs operating under a 1/6 voltage scheme, demonstrating an exceptional rectification ratio of ~ 10^8^ and nonlinearity of 10^5^. **b** Progressive evolution of I–V curves under continuous unidirectional voltage sweeps, illustrating the synaptic conductance modulation. **c** Endurance performance showcasing stable rectification ratio retention through 10^7^ programming cycles, confirming exceptional cycling reliability. **d** LTP and depression LTD characteristics demonstrating synaptic weight modulation in the SRMs under 20-μs programming pulses. **e** Statistical analysis of cycle-to-cycle conductance variations during repetitive weight updates, highlighting the exceptional stability (1.55% variation) at 20-μs pulse intervals. **f** Device-to-device uniformity assessment across a 32 × 32 crossbar array, revealing minimal variation (3.32%) that ensures reliable parallel operation in neuromorphic computing applications. **g** Schematic diagram and flowchart of the hardware-software cooperative anti-attack ADS based on the proposed single-layer SRMs. Reproduced from [51], with permission from Springer Nature

Remarkably, recently, Zhang et al. proposed a groundbreaking advancement in CNN based on SRMs by introducing a Pt/HfO_x_/TiN structure that achieves unprecedented performance metrics, including a RR exceeding 10^8^ and excellent endurance over 10^7^ cycles (Fig. 9a–c) [51]. These achievements are attributed to meticulous engineering of the HfO_x_ layer through rapid thermal annealing, which effectively reduces oxygen vacancy concentrations and optimizes interfacial properties, thereby mitigating sneak path currents and enhancing device uniformity (Fig. 9f). The SRMs exhibit remarkable stability, with minimal device-to-device (3.32%) and cycle-to-cycle (1.55%) variations, making them ideal for scalable crossbar arrays capable of supporting neuromorphic computations at densities exceeding 25.4 terabits (Tb). A pivotal innovation lies in the SRMs’ ability to emulate synaptic plasticity, demonstrating LTP and LTD over 256 analog states with ultra-high precision (Figs. 9d and 7e). This synaptic behavior, coupled with the devices' inherent analog computing capabilities, enables the implementation of hardware-based autonomous driving systems (ADS) based on CNN units (Fig. 9g), showcasing their resilience against adversarial attacks, and maintaining classification accuracies (84.25%) comparable to software models like YOLOv9 (84.34%) even under complex attack scenarios. The proposed SRMs’ intrinsic analog dynamics and localized plasticity further enhance feature extraction and noise suppression, addressing critical challenges in edge computing environments.

Looking ahead, the application of SRMs in CNNs holds transformative potential. Their low-power, high-speed in-memory computing architecture could significantly accelerate convolution operations and matrix multiplications by minimizing data movement and energy consumption. Future research should focus on optimizing SRM-based crossbar arrays to better support weight storage and parallel computations intrinsic to CNNs, enhancing both training and inference efficiency. Additionally, exploring their compatibility with spiking neural networks and attention mechanisms may further boost computational throughput and adaptability. By bridging the gap between hardware capability and algorithmic demand, SRM-accelerated CNNs could become a cornerstone for future energy-efficient and high-performance neural processing systems.

#### Reservoir Computing

**Fig. 10:**
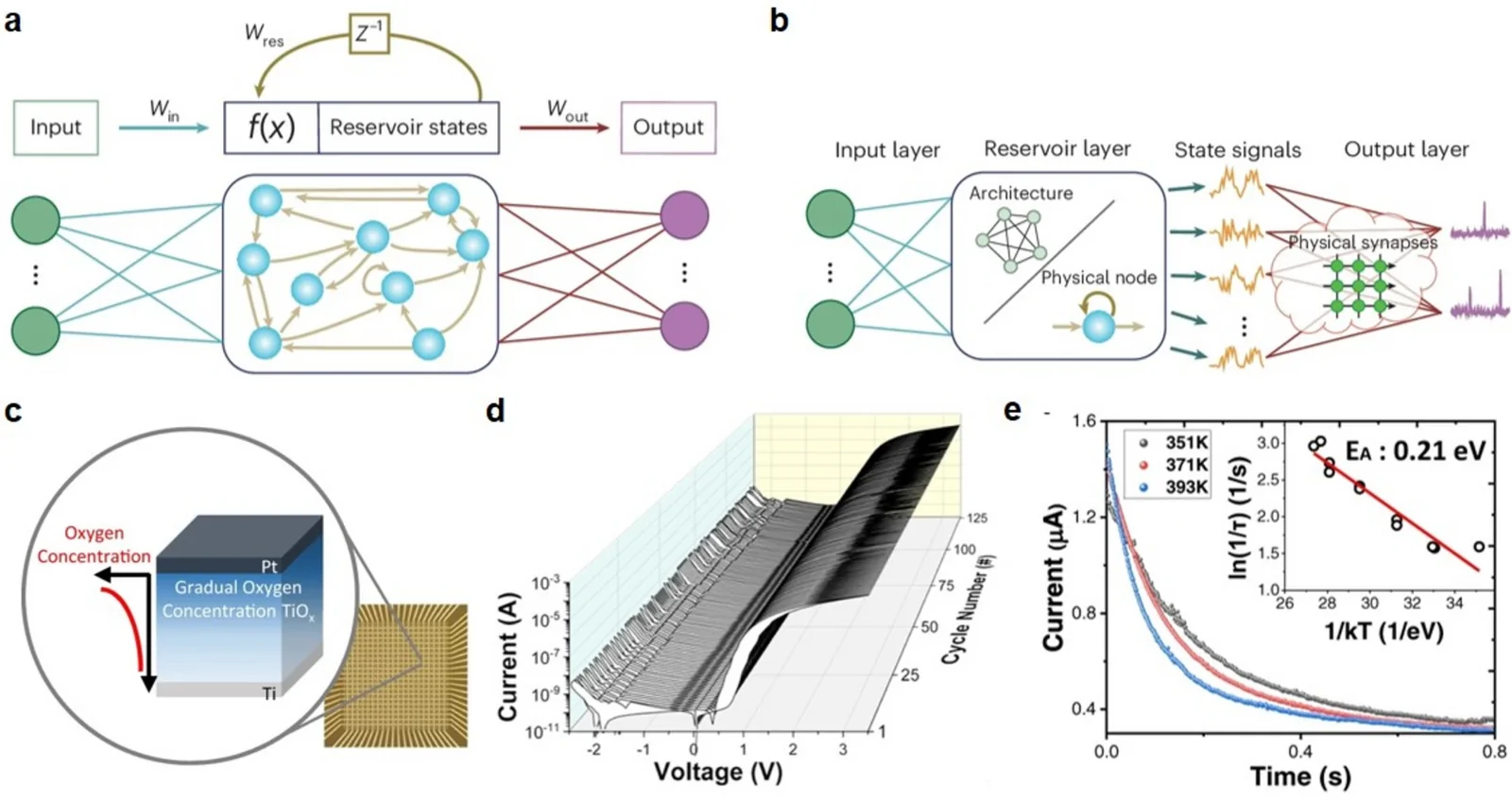
**a** Schematic diagram of digital RC system. *W*_in_, *W*_res_ and *W*_out_ represent input weights, reservoir weights and output weights, respectively, where* Z*^−1^ is decaying processing and *f(x)* is a nonlinear function. **b** Schematic diagram of physical RC system based on emerging memories dominant by SRMs herein. Reproduced from [121], with permission from Springer Nature. **c** Schematic diagram of dynamic volatile SRMs with gradual oxygen concentration in the TiO_x_ layer. **d** DC I–V curves of dynamic volatile SRMs. **e** Decaying nature of the volatile TiO_x_ SRM at different temperatures. Reproduced from [122], with permission from Springer Nature

With the development of Artificial Intelligence (AI), traditional neural networks such as feedforward Deep Neural Networks (DNNs) [123] and Recurrent Neural Networks (RNNs) [124] have been facing many challenges when dealing with complex tasks for many years. Feedforward DNNs are less efficient when dealing with time series data because they lack the ability to effectively model time dependencies [123]. While traditional RNNs are capable of handling time series data, they are prone to the problem of exploding or vanishing gradients during training, leading to difficult training and convergence [125]. Meanwhile, the demand of these traditional neural networks for a large number of training samples and computational resources limits their application in resource-constrained environments such as edge computing. Reservoir computing (RC), an emerging neuromorphic computing paradigm, processes information by exploiting the complex nonlinear behavior of dynamic systems [121, 126]. A fixed and highly dynamic reservoir layer maps the input signal to a high-dimensional space, followed by a simple linear readout layer for output (Fig. 10a), which not only avoids the gradient-related problems of traditional RNNs during the training process, but also ensures fast and high-precision learning with minimal resource requirements [121]. The dynamic behavior of memristors is highly compatible with the requirements of RC, effectively realizing the complex dynamic mapping of the reservoir layer, and at the same time reduce the hardware cost and power consumption [102]. At the same time, the plasticity of memristors enables them to adapt to different input signals and task requirements, further enhancing the flexibility of RC systems [121]. In terms of large-scale integration, RC has relatively low demand for hardware resources and excellent compatibility with existing CMOS technology, providing a stage for dynamic SRMs to play a great role (Fig. 10b) [30, 45, 88, 94, 122, 127].

In 2022, Park et al. experimentally illustrated that the proposed gradient TiO_x_-based SRMs (Fig. 10c) combine neuronal properties, synaptic weight plasticity as well as large RR and NL (Fig. 10d), further confirming the feasibility of SRMs being applied to construct large-scale RC systems for the first time [122]. The decay coefficient of the SRM can be finely tuned by carefully regulating the pulse strategy. Based on the reliable short-term memory effect (Fig. 10e), they constructed a neuromorphic computing system that can efficiently process sequence data, and successfully trained and generated biomedical sequence data (antimicrobial peptides), achieving efficient learning and generation of complex sequences with only a few training parameters. Building on such temporal processing capabilities, the application of SRM-based reservoirs has expanded into cybersecurity. Zhang et al. used dynamic nonvolatile SRMs with dynamic circuitry design to deeply integrate RC and intrusion detection system (IDS) to accurately capture time series patterns in network traffic for fast and accurate detection of anomalies and intrusions [30].

**Fig. 11:**
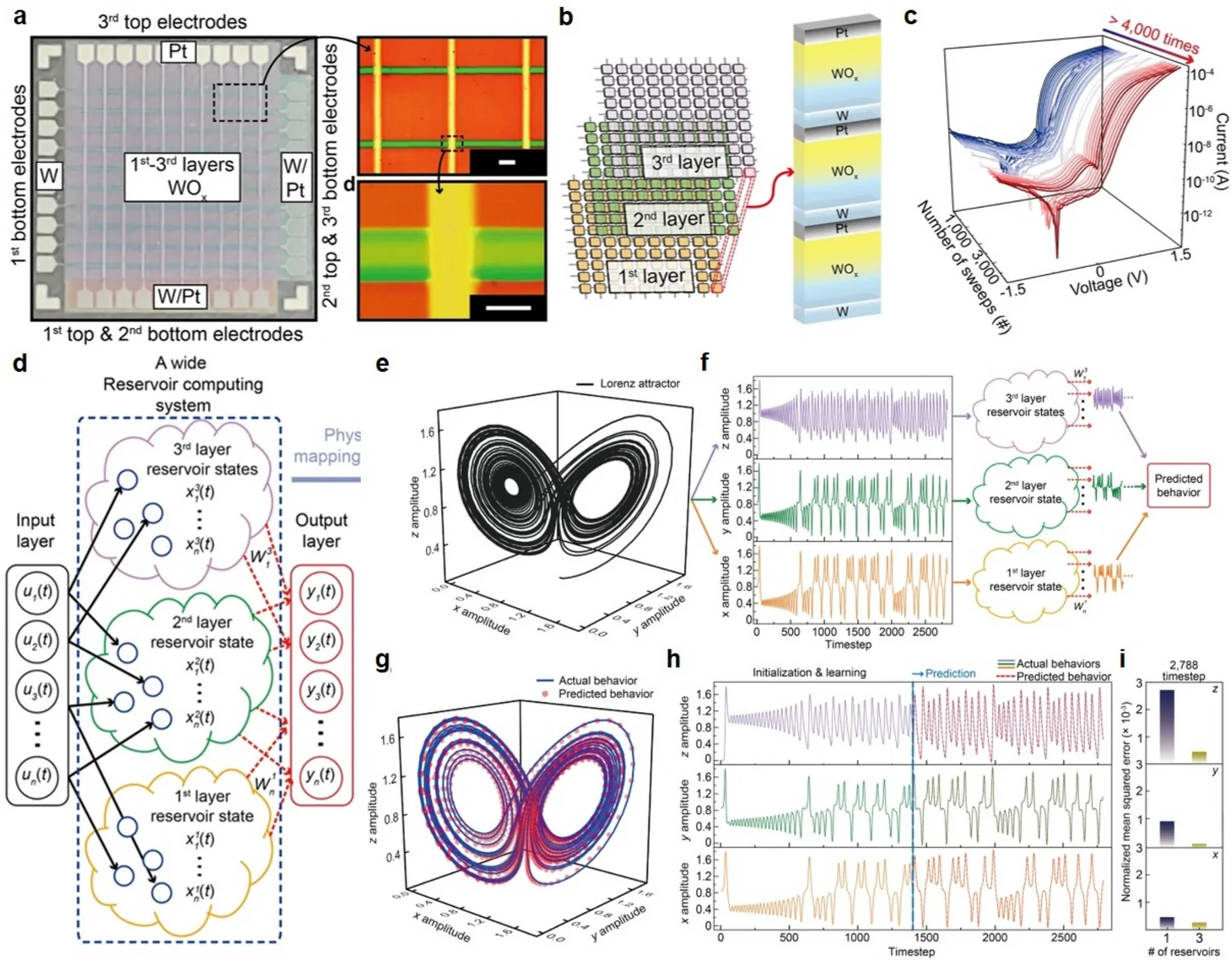
**a** Macroscopic topographic image (left) as well as local magnification (right) of a 3D stacked three-layer 3 × 10 × 10 crossbar array with Pt/WO_x_/W SRMs vertically integrated at each crossing point. **b** Schematic diagram of 3D stack three-layer passive crossbar arrays and SRM cells. **c** DC I–V curves of WO_x_-based SRMs. **d** Schematic diagram of the three-layer RC system. **e** Schematic diagram of a 3D trajectory of Lorenz attractor in the x, y, and z components. **f** Schematic diagram of the decomposition of the one-dimensional Lorentzian attractor to three layers from the three-dimensional Lorentzian attractor as a function of time with the three physical reservoirs used to predict the three-dimensional Lorentzian attractor. **g** Actual and predicted scenarios for the three-dimensional Lorenz attractor. **h** Comparison of actual and predicted behavior of x, y and z components over time. **i** NMSE between actual and predicted behavior of single and multiple reservoirs on x, y and z components. Reproduced from [45], with permission from Springer Nature

Compared to the common single-layer RC systems, by stacking multiple reservoir layers in 3D space, not only the complexity and diversity of the system can be significantly increased, but also local features in time series data can be extracted and processed more effectively. In 2024, Choi et al. designed and fabricated a 3D-integrated multilayer WO_x_ SRM crossover array (Fig. 11a) with a Pt/WO_x_/W SRM cell integrated at each crossover point (Fig. 11b) [45]. This 3D integrated array features forming-free characteristics, high switching uniformity, and over 98% device yield, as well as an ultra-low operating voltage of ~ 0.7 V (compared to advanced SRMs [17, 32]) (Fig. 11c). Based on this, the team designed wide reservoir computing hardware, which expands the feature space by increasing the number of reservoirs (Fig. 11d). Each reservoir can independently process and extract different local features of the time series and map them to different feature spaces, enabling more efficient processing of multivariate time series data. In the biological cell location classification task, the three-reservoir-based RC system achieves 100% classification accuracy using only 15 amniotic cells, while the single reservoir RC system requires 25 amniotic cells to achieve 93% accuracy [45]. In the Lorenz attractor prediction task, Fig. 11e shows the three-dimensional trajectory of the Lorenz attractor, which has a complex dynamic behavior in the x, y, and z directions. To perform the prediction, the researcher decomposed the 3D Lorenz equation into three time-dependent one-dimensional equations (corresponding to the components of the x, y, and z axes, respectively) and input them into a 3D-stacked WO_x_ physical memory array (Fig. 11f). Each physical memory layer processes the chaotic input signals in the corresponding direction to generate separable memory states, subsequently passed to the output layer for learning and prediction. Figure 11g shows the actual Lorenz attractor behavior compared to the predicted behavior after 1400 time steps of learning with remarkably conformity (Fig. 11h) The average normalized mean square error (NMSE) of the three-layer reservoir system is 2.62 × 10^–4^, which is one order of magnitude lower than that of the single reservoir system (NMSE of 1.35 × 10^–3^) (Fig. 11i), indicating that the 3D-stacked structure has higher accuracy and efficiency in predicting the complex dynamic system [45]. This 3D-integrated physical memory array is not only revolutionary and innovative in terms of hardware implementation of RC, but also provides an extremely efficient and compact solution for processing time series data in future AI systems and is expected to play an important role in areas such as large-scale edge computing compatible with CMOS processes.

Recently, the volatile properties of Pt/TaO_x_/TiN SRMs enable dynamic nociceptor-like behaviors such as threshold detection, relaxation, and sensitization, mimicking biological pain responses [81]. The SRM’s uniformity and CMOS compatibility facilitate scalable integration, demonstrated in a 5 × 5 synaptic array and Morse code generation. More importantly, the short-term volatility and nonlinear response of the device are key features that make it particularly suitable for RC system, where such dynamics are harnessed to process temporal information. Additionally, its nonlinear dynamics support reservoir computing, achieving 92.35% accuracy in MNIST recognition, and highlighting the SRMs’ potential for energy-efficient neuromorphic computing, sensory systems, and edge AI applications.

#### Optoelectronic Neuromorphic Computing

**Fig. 12:**
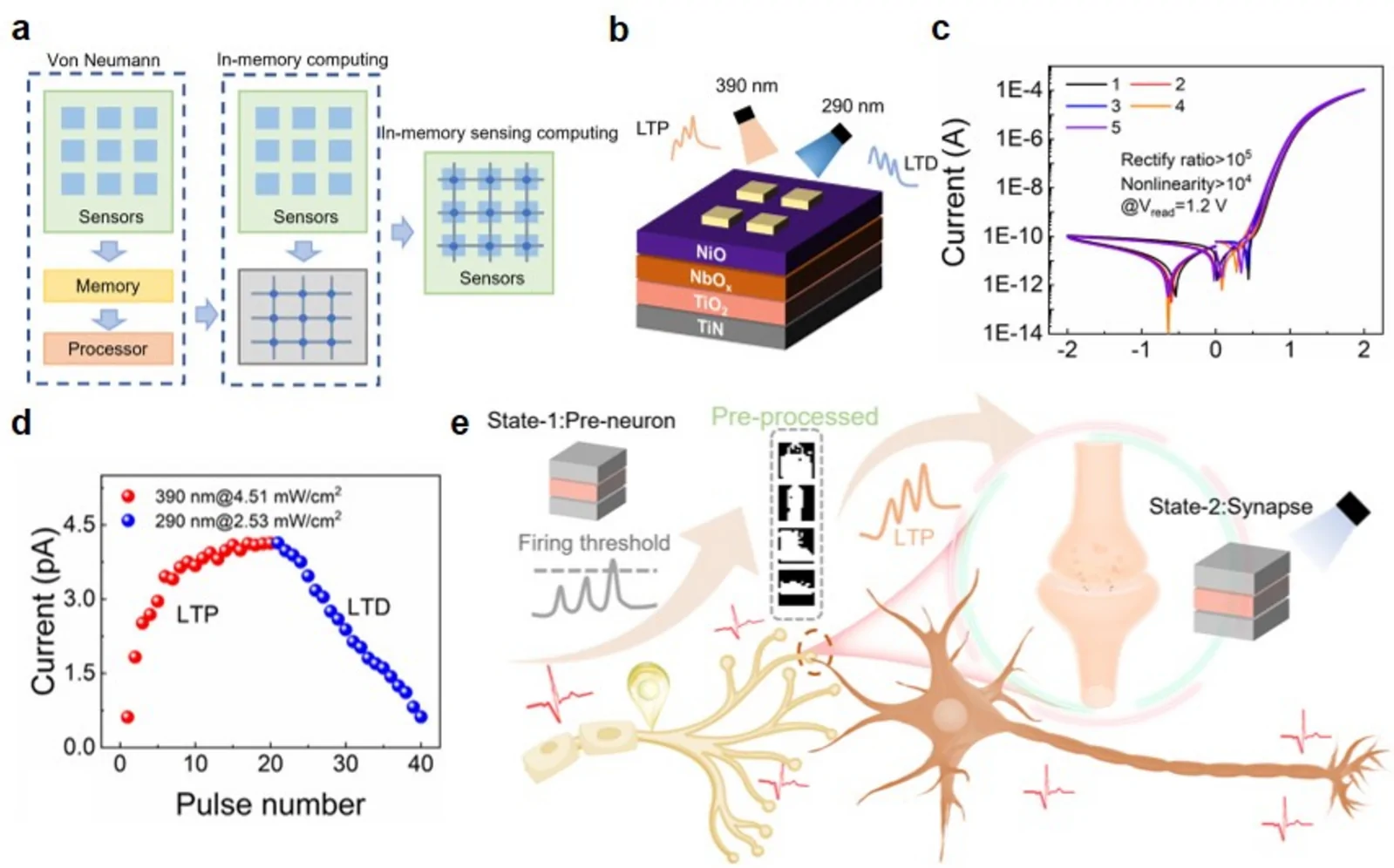
In-sensor computing based on optoelectronic SRMs. **a** Schematic diagrams of the Von Neumann architecture, the traditional architecture with separate sensor and in-memory computing units, and the in-sensor computing architecture. **b** Structure of the optoelectronic SRM and schematic diagram of the dual-wavelength sensing characteristics. **c** DC I–V curves of the optoelectronic SRMs. **d** LTP and LTD characteristics of the optoelectronic SRMs with dual wavelength. **e** Schematic diagram of humanoid brain system with pre-neuron/synapse/post-neuron structure realized based on the proposed optoelectronic SRMs. Reproduced from [37], with permission from American Chemical Society

For traditional architectures, a large amount of raw data collected by sensors from the edge end-side needs to be transmitted to the processor for processing, a process that not only consumes a large amount of energy, but also increases the latency of the system, with the data security receiving a huge threat [7]. In-sensor computing fundamentally subverts the design pattern of separating sensors and processors in traditional computing systems by integrating sensing, memory, and computing functions in the same hardware unit (Fig. 12a) [37]. Further, by simulating the function of biological retina, the hardware based on the in-sensor computing architecture is able to generate adjustable positive/negative photoconductive responses directly after receiving optical stimuli and store them, thus realizing the signal acquisition, conversion, memory, and processing functions similar to those of biological retina [128]. Thus, the development of optoelectronic SRMs and the feasibility of realizing corresponding intersensory computing arrays provide valuable guidance for the future realization of large-scale multimodal intelligent visual information processing systems [129].

In 2024, there are some impressive works about optoelectronic SRMs and further crossbar array for in-sensor computing [37, 86, 90]. Gu et al. found that the optoelectronic SRM synapses based on FTO/TiO_x_/Au structure can simulate multiple functions of synapses, including double-pulse heterogeneous learning rule and learn–forget–relearning rule [90]. Moreover, the group innovatively incorporated optoelectronic SRMs into RC [90]. Lu et al. introduced an all-optical controlled (AOC) optoelectronic SRM array based on NiO/TiO_2_ heterostructure, enabling multilevel storage function with self-rectification characteristics and simulating multiple synaptic functions in the human brain at the same time [86]. This group also proposed a reconfigurable AOC SRM based on Si/SiO_2_/TiN/TiO_2_/NbO_x_/NiO/Ru structure (Fig. 12b), exhibiting high RR and NL to ensure the accuracy of the programming operation (Fig. 12c) [37]. 390 nm and 290 nm UV light was used for the LTP and LTD processes at the synapse, respectively (Fig. 12d). Based on this reconfigurable AOC SRM, they constructed a simple pre-neuron/synapse/post-neuron structure for the realization of an intersensory computational system. By scaling, binarizing, and restructuring a 28 × 28 pixel image from the MNIST dataset to fit a 4-bit pulse input, the device is able to convert pixel points of different gray levels into pulse signals with different voltage amplitudes and pulse widths, which are then fed into the pre-neuron. If the input signal is strong enough, the neuron function of the device can output a binarized image (Fig. 12e) [37]. These results indicate that the proposed AOC SRMs have the potential to serve as efficient hardware components in future intelligent sensing systems.

#### Self-Supervised Learning

In terms of supervised learning, models usually rely on large-scale labeled datasets for training, which are quite demanding in terms of labeling and susceptible to problems such as overfitting, spurious correlation, and adversarial attacks [130]. Whereas unsupervised learning is costly and usually lacks direct guidance on downstream tasks, its learned feature representations may not fully match the needs of a particular task, thus having limitations in task migration and generalization capabilities [131]. Unlike traditional supervised and unsupervised learning, self-supervised learning automatically obtains “labels” from data and learns the intrinsic structure of the data by predicting parts of the data, which is not only capable of dealing with large-scale unlabeled data, but also capable of learning feature representations that are broadly applicable to downstream tasks [132]. In short, self-supervised learning utilizes the data itself as a supervisory signal without manually labeling the data, which significantly improves the processing efficiency and generalization ability, and brings a new breakthrough to the field of deep learning. Therefore, passive crossbar arrays based on SRMs show great potential in processing large-scale unlabeled data and improving model generalization ability [46].

**Fig. 13:**
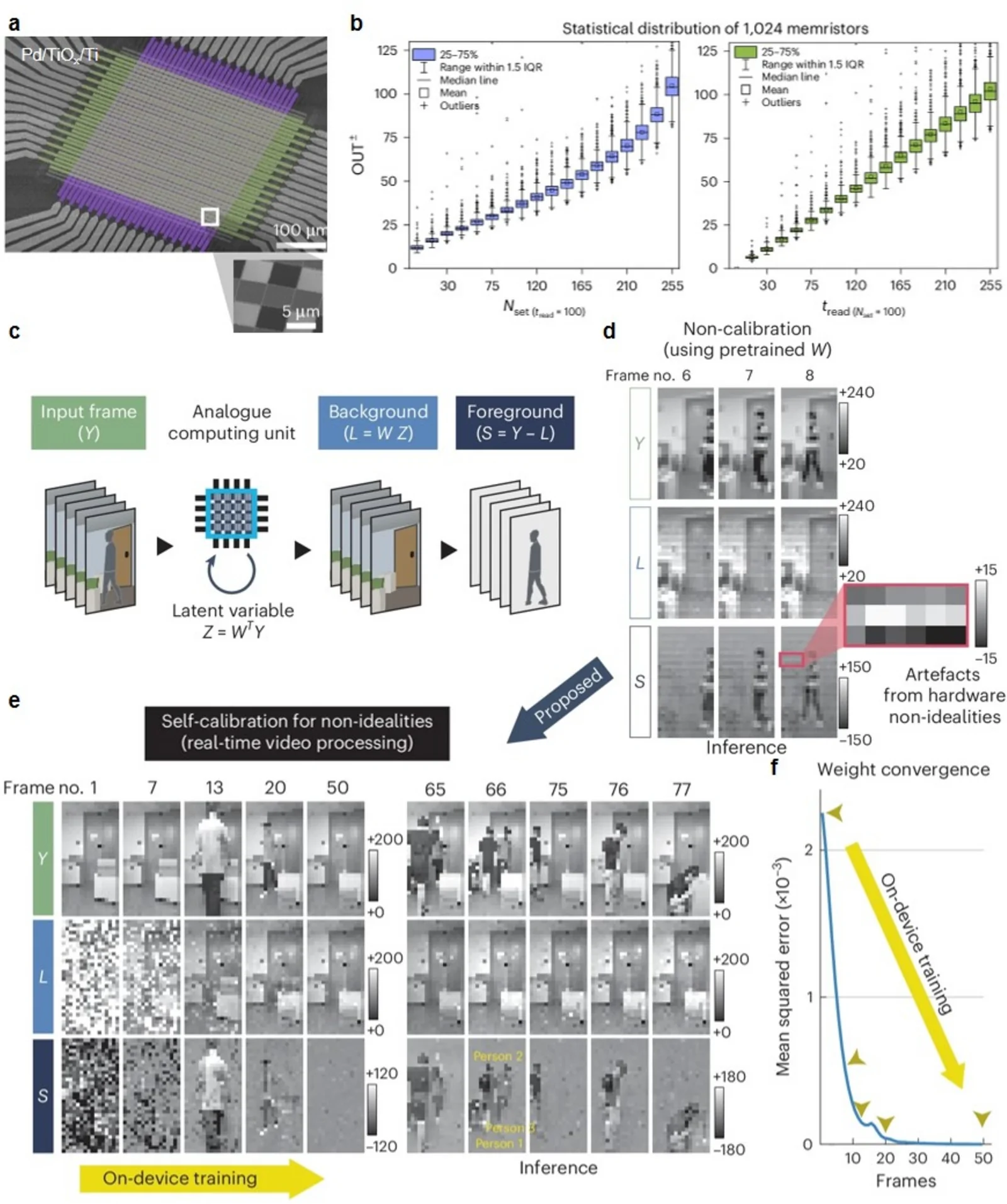
Self-supervised video processing with self-calibration based on analog SRMs. **a** SEM image of passive crossbar array consisting of Pd/TiO_x_/Ti SRMs. **b** Statistical distribution of the analog-to-digital converter (ADC) output that is automatically quantized based on the number of set pulses (*N*_set_) and the number of treadmills for all 1024 Memristors when keeping *t*_set_ (left) and *N*_set_ (right) at 100. **c** Schematic diagram of the self-supervised analog computing unit for real-time video processing. **d** 32 × 16 video data output obtained by analog computation without self-calibration. **e** Real-time video processing with on-device training from untrained weights (left) and real-time inference after on-device training (right). **f** The corresponding mean square error for each frame. Reproduced from [46], with permission from Springer Nature

In early 2025, Jeong et al. [46] broke new ground by developing a self-supervised learning algorithm for foreground and background separation of videos on an SRM-based hardware platform (Fig. 13a). This algorithm decomposes video frames through an optimization problem, where the background is considered as a low-rank projection of the input data, while the foreground is regarded as a sparse component. The algorithm is trained directly on hardware via gradient descent method without the need for a pre-training process, allowing the hardware platform to adapt to frequently changing information environments and to self-optimize through continuous training. Figure 13b shows the statistical distribution of the unsigned 8-bit ADC outputs (OUT ±) of all SRM cells in the developed array, demonstrating that all the memristor devices operate reliably in the analog domain and that the cells perform operations without the need for compliance currents and compensation algorithms. The low-rank component (L, background) is obtained in the proposed self-supervised real-time video processing architecture by performing two rounds of matrix multiplication operations on the raw video data (Y, input frame) using the same computational unit. The latent variable (Z) is computed from the first round of matrix multiplication and the sparse component (S, foreground) is obtained by subtracting L from Y (Fig. 13c). By performing direct on-device training, the system is able to automatically learn and calibrate the non-idealities of the hardware (Fig. 13d). In the experiment, the system separates foreground and background in real time at about 0.7 frames s^−1^, and after about 28 frames of training, the system successfully converges (Fig. 13f) and is able to accurately separate foreground and background in the video (Fig. 13e) [46]. To sum up, since memristors introduce some non-ideal factors such as inter-device variations and inter-week variations; these factors may affect the accuracy of the computed results. By training directly on the proposed SRM hardware, the self-calibration algorithm is able to automatically learn and adapt to these non-ideal factors, thus achieving accurate video separation without relying on external compensation algorithms.

### Hardware Security

In today’s highly interconnected information technology era, hardware security faces many challenges, such as data leakage, hardware Trojans, and theft of chip design intellectual property [30]. To cope with these challenges, hardware security technologies are constantly evolving. Among them, Physical Unclonable Function (PUF) generates unique and lightweight “digital fingerprints” by capturing small differences between chip devices [100]. For the beyond-CMOS computing paradigm, PUFs are important because they provide a low-cost, highly secure and robust solution for key storage, device authentication and IP protection [133]. For example, silicon-photonic PUFs utilize the unique attributes of silicon photonic technology to provide direct security authentication for optical communication systems by enabling the integration of optical functionality in a standard CMOS process [134]. By eliminating crosstalk between array units, SRMs can improve the read accuracy and stability of PUFs, thereby enhancing the robustness of PUFs in the face of complex environments and attacks. At the same time, SRMs also facilitate the ability of PUFs to achieve higher security and sophistication in a smaller chip area, a lightweight that is particularly important for resource-constrained IoT devices and edge computing devices [135–138].

**Fig. 14:**
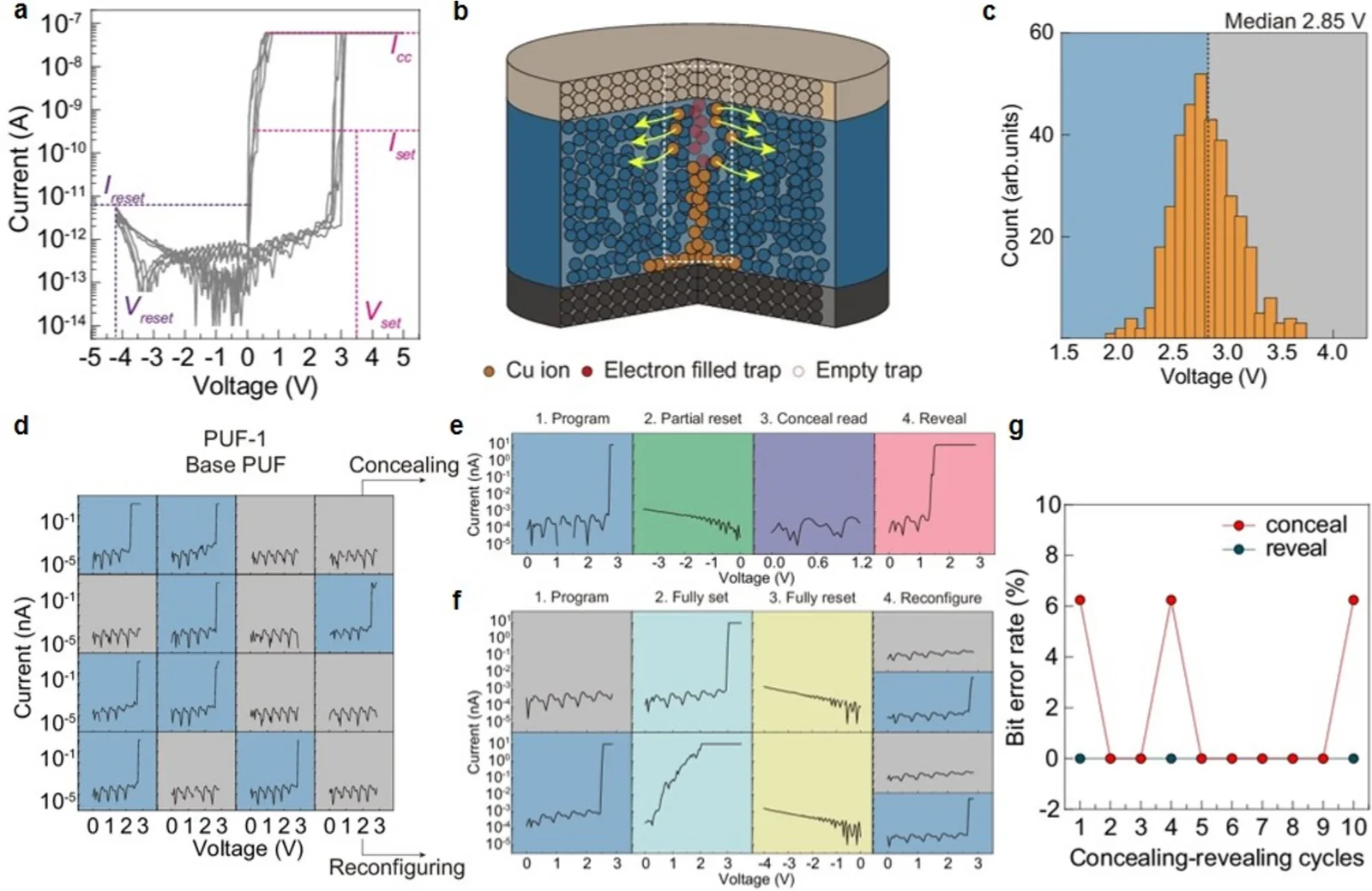
PUF implementation by using tunable memristors with self-rectification effect. **a** DC I–V curves of the SRMs with specific component. **b** Conducting mechanism of the device when presenting the self-rectification effect. **c** Entropy source for PUF implementation. **d** 4 × 4 PUF map based on random switching. **e** Concealing process of the proposed PUF implementation based on SRMs. **f** Reconfiguring process of the proposed PUF implementation based on SRMs. **g** Bit error rate of ten concealing-revealing cycles. Reproduced from [47], with permission from Springer Nature

Woo et al. proposed a PUF based on ion-migration-driven SRMs in a Cu_0.3_Te_0.7_/HfO_2_ (CuTeHO) structure with not only reconfigurability but also concealability [47], which had not been realized in previous memristor-based PUF systems [14, 100, 139]. They achieved the transition from volatile threshold switching behavior to nonvolatile resistive switching behavior by adjusting the copper concentration (*x*) of the Cu_x_Te_1-x_-based memristor. At *x* = 0.3, the CuTeHO-based memristor exhibits nonvolatile and self-rectification behavior (Fig. 14a). Copper ions migrate from the CuTeHO electrode to the HfO_2_ dielectric layer under the action of an electric field, forming conductive filaments. The stability of these filaments depends on their surface curvature and the thickness of the filaments. At *x* = 0.3, the filaments have less surface curvature and are more stable (Fig. 14b). Also the filling and de-filling process of the trap state affects the stability of the conducting filaments, which in turn shapes the rectification behavior. The SET voltage distribution of the CuTeHO-based SRM has a random nature (Fig. 14c), making it possible for each SRM to switch to a LRS or to remain in a high-resistive state (HRS) under the same bias voltage, which generates the distinctive PUF response (Fig. 14d). The unique concealability stems from the fact that applying a partial RESET voltage to all SRMs converts them to a partial HRS, at which point the resulting response mapping is scrambled to hide the PUF data (Fig. 14e). The critical reconfigurability is achieved by assigning a new and random SET voltage to the SRMs through the RESET process after ensuring that all SRMs are in the LRS (Fig. 14f). Finally, Fig. 14g confirms the reliability of the concealability feature of this SRM-based PUF [47]. Compared to earlier SRM-based PUFs, this PUF is a breakthrough in both energy efficiency and security [135–138]. Besides, the veritical SRM with outstanding computational and area efficiency exploits the inherent device-to-device variations in the HRS of 3-layer Pt/Ta_2_O_5_/Al-doped HfO_2_/TiN devices to generate unique and reproducible PUF keys, where the small cycle-to-cycle variation ensures reliable key regeneration, while the concealment feature enhances security by hiding keys when not in use [140]. This integrated approach combines PUF generation and encryption in a single platform, offering a compact, energy-efficient, and scalable solution for secure edge computing applications. Recently, a novel SRM-based PUF model [141] and a novel scheme for reliable encryption of high-resolution images [142] were proposed that achieved high memory density with mitigating sneak path currents, demonstrating significant improvements in uniqueness, uniformity, and reliability for hardware security applications further.

The works seamlessly integrate SRMs with reconfigurable, concealable PUFs, facilitating the move toward extreme lightweighting of PUF and even other hardware security applications.

## Potential Challenge and Outlook

**Fig. 15:**
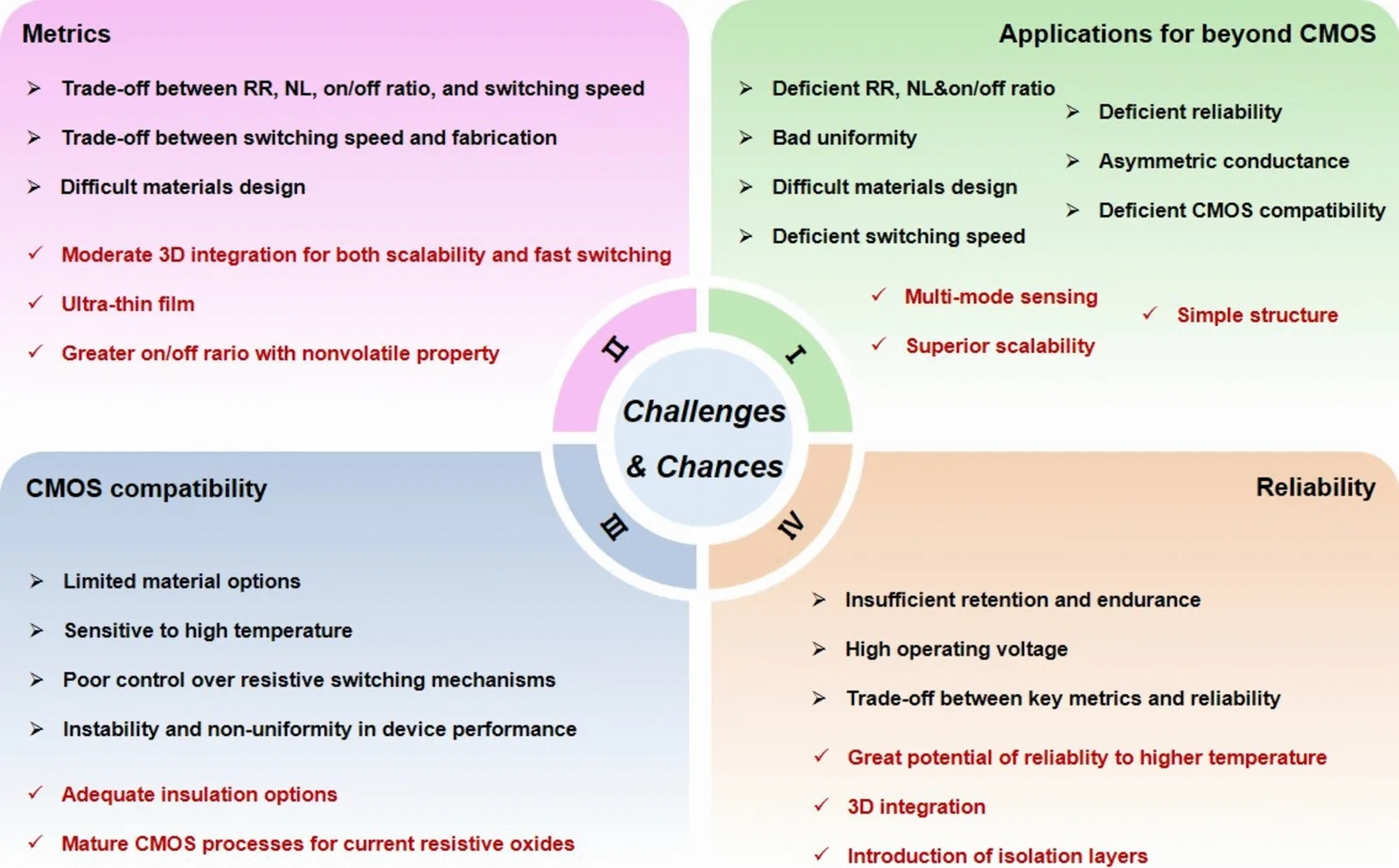
Major challenges and opportunities for the future development of SRM

In the last section, we provided a comprehensive overview of the current state of research on SRMs and explored in depth the potential of SRMs in beyond-CMOS computing paradigms, analyzing their compatibility with CMOS processes and their impact on novel computing architectures. Although SRMs show great potential and wide application perspectives in beyond-CMOS computing paradigms including in-memory computing, neuromorphic computing, and hardware security, they all face serious challenges in terms of fabrication process, device performance, and application generalizability inevitably. In this section, we will provide a comprehensive discussion on the further development of CMOS compatibility of SRMs as well as the challenges, potential solutions, and opportunities for the design of future high-performance, low-power computing architectures (Fig. 15).

### Application for Beyond CMOS

#### In-Memory Computing

Regarding in-memory computing, encompassing standard VMM [34], logic [57], and content addressing [109], 10^3^ RR and NL have adequately met the requirements of the associated proof-of-concept demonstration. However, the emerging in-memory computing architectures, led by sparse matrix multiplication, have demonstrated that to achieve high accuracy (lowest possible read error rate) it is necessary to significantly improve RR, NL, on/off ratio, and switching speed instead of compromising on trade-off to guarantee a virtually crosstalk-free VMM. As mentioned above and in conjunction with Table 1, these three parameters significantly affect the scalability of the SRM, which in turn deteriorates the application up-limitation of the devices for in-memory computing. Subsequently, one of the most important factors is the fact that it is difficult to achieve 100% yields for passive crossbar arrays at this stage of the fabrication process for advanced SRMs, and the yields of devices obtained from non-CMOS-compatible processes are even worse [40]. This would significantly result in unnecessary read errors, making VMM much less reliable. However, following extensive research conducted in recent years [34], Reference [32] has developed passive crossbar arrays based on SRM with a yield of 100%, offering valuable insights for enhancing the yield of more advanced SRMs in the future.

Notably, in the field of in-memory computing, the measurement of low on-current (particularly < 1 μA) in SRMs presents significant challenges in pulse mode, arising from the combination of the small current levels and the transient nature of pulse measurements, which can introduce severe noise and measurement inaccuracies [143]. In pulse mode, the transient nature of the current can lead to significant noise, making it difficult to accurately measure low on-current levels [144]. The short duration of the pulses means that the current has limited time to stabilize, and any noise present in the system can be magnified, which comes from various sources, including thermal noise, shot noise, and power supply noise. Furthermore, the difficulty in measuring low on-current in pulse mode has direct implications for the design of low-power consumption in-memory computing circuits, where accurate current measurement is essential for optimizing the energy efficiency of these circuits [145]. On the contrary, inaccurate measurements can lead to overestimation or underestimation of the current, which can further affect the overall power consumption and performance of the system. For example, if the on-current is underestimated, the circuit may not provide sufficient current to perform the desired operations, leading to performance degradation. Conversely, if the on-current is overestimated, the circuit may consume more power than necessary, reducing energy efficiency and even breakdown of the whole system.

To address these challenges, several strategies can be employed. One approach is to use high-precision current measurement techniques, such as current–voltage measurements with low noise amplifiers and high resolution digital-to-analog converters. These techniques can help reduce the impact of noise and improve the accuracy of low-current measurements. Additionally, using pulse shaping techniques to extend the duration of the pulses can help stabilize the current and reduce the impact of transient noise [146].

#### Neuromorphic Computing

In relation to neuromorphic computing, the ANN accelerator and RC system utilizing SRMs have reached maturity for image recognition and time series signal classification implementation [32, 45, 122]. By contrast, the hardware implementation of CNNs using SRMs-based hardware faces a number of challenges. In terms of the device level, SRM-based passive crossbar arrays suffer from low yields and excessive device-to-device and cycle-to-cycle variations due to immature fabrication processes, which in turn affects the overall computational accuracy and stability. In terms of convolution operation, the key convolution operation in CNN requires sliding operation on different input patches, which is usually a sequential process, leading to speed mismatch between the convolver and the passive crossbar array used for the fully connected VMM, which reduces the computational efficiency. In terms of training, traditional pure in situ training requires highly complex operations to back-propagate the target derivatives to determine the weight updates, while training of complex memristor-based deep neural networks becomes challenging due to the properties of the device such as nonlinearity and asymmetric conductance tuning. In addition, when transferring the weights obtained from offline training to the conductances of the SRMs, defective devices of the hardware, parasitic line resistances and capacitances, etc. can blur the weights and degrade the system performance [120]. For system integration, the integration and scalability of SRM arrays are key issues. Scalability can be ensured by optimized RR, NL, and on/off ratio, while integration depends on the maturity of 3D integration technology. In the future, the weights of the fully-connected layers are adjusted to compensate for the non-ideal characteristics of the device by performing local training in an SRM-based hardware system. This hybrid training approach combines the efficiency of software training with the adaptability of hardware training and is able to significantly reduce the hardware resource requirements during training while maintaining high recognition accuracy [120].

#### Hardware Security

In terms of hardware security, device-to-device variations in passive crossbar arrays enable a rich source of entropy for the construction of secure primitives. However, cycle-to-cycle variation remains a great challenge that limits their security. One of the current state-of-the-art PUFs has ultra-low cycle-to-cycle variations with BER remaining at < 6.78 × 10^–6^ after 10^8^ read cycles, i.e., no errors in 144 Kb arrays [147]. After breaking, the performance bottlenecks of RR, NL, and on/off ratio, need to take extra consideration of their relationship with reliability (retention and endurance) as a trade-off challenge. Besides, even though existing memristor-based true random number generators (TRNGs) have been able to achieve extremely high throughput [148], the energy efficiency and area efficiency of large-scale arrays based on 1T1R cells cannot be compared with passive crossbar arrays based on SRM cells of the same size [118]. In order to meet the demand for data protection in the Big Data era, TRNG-oriented ultra-large-scale SRM arrays are likely to become mainstream. Last but not least, homomorphic encryption (HE) enables data to be “counted but not seen,” essentially securing data processing as well as communication [149]. In 2024, the first case of memristor implementation of HE was presented, which demonstrated that HE requires particularly high analog characteristics and uniformity of arrays [149], putting further pressure on SRM development [150].

#### In-Sensor Computing

In terms of in-sensor computing, optoelectronic SRM units with sensing capabilities have initially demonstrated the feasibility of constructing and refining an ultra-large-scale retinal-like neuromorphic system aiming for much higher sensitivity and parallel processing speed [37, 90]. However, according to the current researches, the processing data magnitude is low, the task is relatively simple, and the devices only have simple sensing or storing performance, which is still far away from the real sense of all-in-one hardware [37, 90]. We have discussed the challenges and solutions for SRM-based in-memory computing applications in the previous section, and there are still many problems to be solved in sensing. The most notable ones remain uniformity and stability, where the lack of the former will result in the information captured by the sensing layer not being effectively processed in situ, and the lack of the latter will diminish the utility of the hardware. Subsequently, designing a suitable material system is a prerequisite for constructing the basic unit of sensing, memory, and computing [151]. Sensing, memory and computing units are combined based on different material combinations, device structures, and heterogeneous integration techniques, and it is very challenging to integrate these three functions into a single device while taking into account the rectification characteristics. The availability of materials for different sensing sources (chemical, radiation, temperature, pressure) is still very limited, so the development of SRM-based multimodal (visual, tactile, auditory, olfactory, etc.) interoceptive computing systems still has a long way to go.

### Comparison with Other Beyond-CMOS Technologies

In the quest for beyond-CMOS technologies, SRMs have garnered significant attention due to their unique combination of intrinsic diode-like rectification and non-volatile memory capabilities. Furthermore, to fully appreciate the potential of SRMs, it is essential to compare them with other emerging technologies that are also explored for applications such as in-memory computing, neuromorphic computing, and hardware security.

#### Spintronic Devices

Spintronic devices, which leverage the spin of electrons to store and process information, offer high endurance and fast switching speeds, making them suitable for high-speed memory applications [152]. These devices, such as magnetic tunnel junctions used in spin transfer torque magnetic random access memory, exhibit non-volatile memory and low-power operation [153]. However, the fabrication of spintronic devices often requires sophisticated processes and materials, which can increase manufacturing complexity and cost. And scaling down spintronic devices to smaller dimensions can be challenging due to the need to maintain magnetic stability and avoid interference between adjacent devices. Furthermore, spintronic devices can generate significant heat during operation, necessitating advanced thermal management solutions to maintain performance and reliability [154].

#### Quantum Computing Elements

Quantum computing elements, such as superconducting qubits and trapped ions [155], exploit quantum phenomena to perform computations and offer the potential for exponential speedup in solving certain complex problems. These technologies can handle complex problems that are infeasible for classical computers [156], opening up new possibilities in fields like cryptography, materials science, and machine learning. However, quantum bits are highly sensitive to environmental noise, leading to high error rates and short coherence times, which limit the reliability and duration of quantum computations. Many quantum computing elements require extremely low-temperature environments to maintain their quantum states, necessitating complex and expensive cooling systems. Scaling up quantum systems to a large number of qubits while maintaining low error rates and implementing effective error correction is a significant challenge [27].

#### Other Emerging Technologies

Beside spintronics and quantum computing, several other emerging technologies are explored for beyond-CMOS applications. For instance, memristive devices based on different material systems, such as two-dimensional materials like molybdenum disulfide (MoS_2_) and graphene, offer unique advantages. These materials exhibit high carrier mobility, tunable bandgaps, and excellent mechanical flexibility, making them suitable for flexible and wearable electronics [157–159]. Moreover, 2D materials can be integrated into van der Waals heterostructures, enabling the development of novel devices with enhanced performance [160]. However, the fabrication of 2D material-based devices often requires precise control over the material synthesis and layer stacking, which can be technically challenging and costly. And the scalability and uniformity of 2D materials in large-scale arrays remain significant challenges [161]. Another promising technology is molecular electronics, which involves using organic molecules as the active components in electronic devices. Molecular electronics can offer high scalability and low-cost fabrication, making them attractive for large-area and flexible electronics applications [162]. But the performance of molecular devices can be highly variable due to the inherent randomness in molecular structures and the difficulty in achieving uniform molecular alignment.

Phase change memory (PCM) is another emerging technology that leverages the reversible phase transition of chalcogenide glasses between amorphous and crystalline states to store information [163]. PCM devices offer high write speeds and good scalability, making them suitable for high-performance memory applications [164, 165]. The ability to switch between states rapidly enables fast data write operations, which is crucial for applications requiring quick data updates. Nonetheless, PCM devices typically require high power to switch between states, which can limit their energy efficiency. What’s more, PCM devices may have limited endurance due to the physical changes in the material during switching, which can lead to degradation over time, with the fabrication of PCM devices often requiring sophisticated processes and materials, which can increase manufacturing complexity and cost [163].

In contrast, SRMs combine several desirable properties that set them apart from other beyond-CMOS technologies. Their intrinsic diode-like rectification and non-volatile memory capabilities enable high-density integration without external selectors, simplifying design and reducing power consumption. This is particularly advantageous for applications such as in-memory computing and neuromorphic computing, where high-density and low-power operation are critical.

## Conclusions

Self-rectifying memristors (SRMs) have emerged as a viable candidate for beyond-CMOS computing systems, providing a distinctive combination of nonlinearity, tunable conductance, rapid switching, and little power consumption. Their capacity to inhibit sneak path currents via unidirectional conductivity further amplifies their potential for scalable in-memory computing, neuromorphic computing, and hardware security applications. This review has systematically analyzed the working mechanisms, characteristics, and applications of SRMs, highlighting their compatibility with CMOS processes and their impact on novel computing paradigms. Despite significant progress, several challenges remain in the development of SRMs for large-scale integration and practical deployment. These include optimizing rectification ratios, nonlinearity, on/off ratios, and switching speeds, while maintaining high reliability and CMOS compatibility. Additionally, addressing device-to-device variability, improving yield rates, and ensuring scalability in passive crossbar arrays are critical for realizing the full potential of SRMs in beyond-CMOS applications. Future research should focus on developing advanced material systems and device structures that can achieve superior performance metrics while maintaining compatibility with existing CMOS processes. Exploring novel applications such as in-sensor computing and self-supervised learning will further expand the scope of SRMs in next-generation information technology. Through interdisciplinary collaboration and inventive innovation, SRMs are set to significantly influence the future of high-performance, low-power computing architectures.
